# Supplementary material for: A microscopic Kondo lattice model for the heavy fermion antiferromagnet CeIn3
Source: Nat Commun. 2023 Dec 12;14:8239. doi: 10.1038/s41467-023-43947-z (PMC10716136; doi:10.1038/s41467-023-43947-z)
Supplement: Supplementary file 1 — Supplementary Information [file 41467_2023_43947_MOESM1_ESM.pdf]

Supplementary information for
A microscopic Kondo lattice model for the heavy fermion antiferromagnet $CeIn_3$

W. Simeth[1], Z. Wang[2,3,*], E. A. Ghioldi[2], D. M Fobes[4], A. Podlesnyak[5], N. H. Sung[4], E. D. Bauer[4], J. Lass[6], S. Flury[1,7], J. Vonka[1], D. G. Mazzone[6], C. Niedermayer[6], Yusuke Nomura[8], Ryotaro Arita[8,9], C. D. Batista[2,10], F. Ronning[4], and M. Janoschek[1,4,7]

[1]*Laboratory for Neutron and Muon Instrumentation, Paul Scherrer Institute, Villigen PSI, Switzerland*

[2]*Department of Physics and Astronomy, The University of Tennessee, Knoxville, TN, 37996, USA*

[3]*School of Physics and Astronomy, University of Minnesota, Minneapolis, Minnesota 55455, USA*

[4]*Los Alamos National Laboratory, Los Alamos, NM 87545, USA*

[5]*Neutron Scattering Division, Oak Ridge National Laboratory, Oak Ridge, Tennessee 37831, USA*

[6]*Laboratory for Neutron Scattering and Imaging, Paul Scherrer Institute, Villigen PSI, Switzerland*

[7]*Physik-Institut, Universität Zürich, Winterthurerstrasse 190, CH-8057 Zürich, Switzerland*

[8]*RIKEN Center for Emergent Matter Science, Wako, Saitama 351-0198, Japan*

[9]*Department of Applied Physics, The University of Tokyo, Hongo, Bunkyo-ku, Tokyo 113-8656, Japan*

[10]*Quantum Condensed Matter Division and Shull-Wollan Center, Oak Ridge National Laboratory, Oak Ridge, TN, 37831, USA and*

[*]*Present address: Center for Correlated Matter and School of Physics, Zhejiang University, Hangzhou 310058, China*

Abstract

This supplementary information contains details on the theoretical and experimental methods of our study. First, we provide the details for our electronic structure calculations for $CeIn_3$ performed via density functional theory alongside details for our tight-binding model. Subsequently, we elaborate on the multi-orbital periodic Anderson model, which is the starting point for our microscopic model, and show how we derive the effective low-energy Hamiltonians. In the second part we provide details on the experiments. The experimental methods part starts with an account on the sample preparation and quality. After a description of the instrumental setup used for the inelastic neutron scattering experiments, we discuss the data analysis used to determine the dispersion of magnetic excitations. Subsequently, the experimentally determined magnon dispersion for $CeIn_3$ is compared with the results from the microscopic model. Finally, the electronic band structure obtained via density functional theory is compared to electronic band structure of $CeIn_3$ obtained via angle-resolved photo emission spectroscopy (ARPES) experiments.

I. MICROSCOPIC MODELS

The starting point for all materials specific models of f-electron systems is the multi-orbital periodic Anderson model (MO-PAM) $\mathcal{H}_{\text{MO-PAM}}$ presented in Eq. (1) of the main text. To obtain the parameters of $\mathcal{H}_{\text{MO-PAM}}$, we derive a tight-binding model based on density functional theory (DFT) that accurately captures the electronic structure of the conduction bands near the Fermi level E_F, and yields the hybridization between these bands and the $4f$-orbitals. We first performed the DFT band structure calculation for $CeIn_3$ using the QUANTUM ESPRESSO package [1] with the experimental lattice parameter: $a = 4.689$ Å [2]. We employed fully relativistic relativistic projector augmented-wave (PAW) pseudopotentials with the Perdew-Burke-Ernzerhof (PBE) exchange-correlation functional, which are available in the PSlibrary [3]. The calculation was performed with an $11\times11\times11$ $\mathbf{k}$-mesh and an energy cutoff of 175 Ry for the wave function and 700 Ry for the charge density.

To obtain a realistic tight-binding Hamiltonian, 50 Wannier functions (25 orbitals times 2 for spin) were constructed using the Wannier90 package [4], in which the projections of Ce s-, Ce d-, Ce f-, In s-, and In p-type orbitals were employed. We used an inner energy window of [-1.5 eV : $+2$ eV] with respect to the Fermi level (E_{F}) to accurately reproduce the band structure around E_{F}. The excellent agreement between our tight-binding model and the DFT computed band structure is shown in Fig. S1.

Notably, the DFT calculation and the resulting tight-binding model assume that the f-electrons are itinerant, whereas strong correlations will be responsible for localizing them. To illustrate that the bare conduction electron bands remain practically unchanged irrespective of the treatment of the f-electrons, in Fig. S2 we present the DFT band structure with the f-electrons localized in the core. We also include the bands obtained from the tight-binding model with itinerant f-electrons when setting the f-c hybridization to zero $V_{\mathbf{k},\sigma\mathbf{s}} = 0$. The good agreement illustrates that the bare conduction electron bands are insensitive to the treatment of the f-electrons. Furthermore, the quasi-flat nature of the resulting f-bands [bandwidth smaller than $0.03\,\text{eV}$ ($0.07\,\text{eV}$) for the $J = 5/2$ ($J = 7/2$) subbands] (shown in Fig. 2 of the main text) indicates that the f-f hopping is negligible due to the small extension of the $4f$-orbitals. We note that the electronic band structure obtained in this way is also consistent with measurements of the electronic structure as demonstrated in

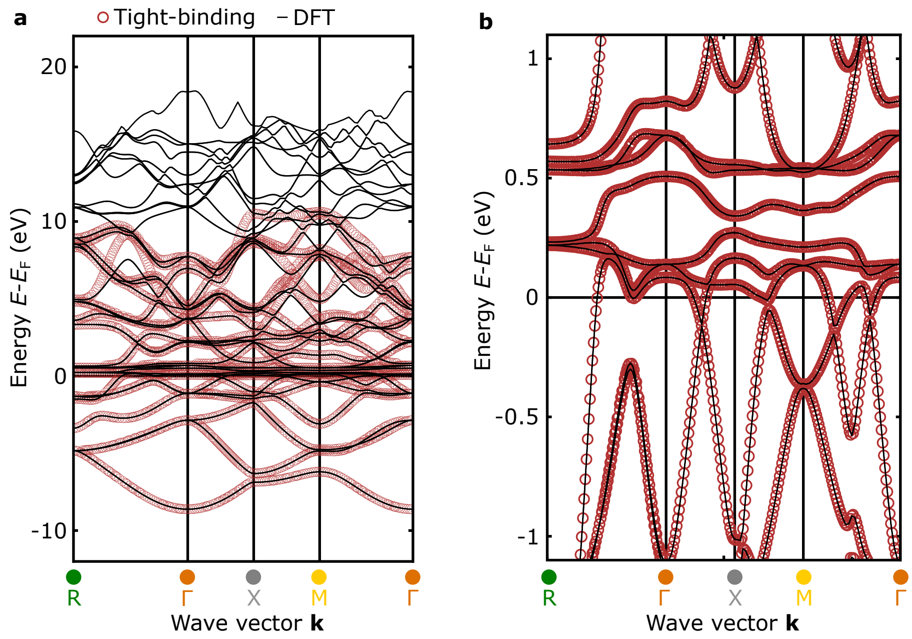

FIG. S1. **Electronic band structure of $CeIn_3$.** Comparison of the DFT calculated band structure (solid lines) and the electronic structure of our 25-band tight-binding model (symbols). **a** Comparison including all the bands. **b** Comparision near E_F.

section X.

From the first-principles calculations, we obtain the tight-binding model that includes 25 orbitals per spin. The low-temperature magnetic properties of $CeIn_3$ at zero field are dominated by the low-energy Ce $4f$ Γ_7 doublet that results from diagonalizing the single-site f-electron Hamiltonian [second term of Eq. (1) in the main text]:

$$|\Gamma_7;+\rangle \equiv |\uparrow\rangle = \sqrt{\frac{1}{6}}\left|j=\frac{5}{2}, m_j=\frac{5}{2}\right\rangle - \sqrt{\frac{5}{6}}\left|j=\frac{5}{2}, m_j=-\frac{3}{2}\right\rangle, \tag{S1a}$$

$$|\Gamma_7;-\rangle \equiv |\downarrow\rangle = \sqrt{\frac{1}{6}}\left|j=\frac{5}{2}, m_j=-\frac{5}{2}\right\rangle - \sqrt{\frac{5}{6}}\left|j=\frac{5}{2}, m_j=\frac{3}{2}\right\rangle. \tag{S1b}$$

By taking the limit of infinite intra-atomic f-f Coulomb repulsion which eliminates the f^2 configurations, we project $\mathcal{H}_{\text{MO-PAM}}$ into the low-energy subspace generated by the Γ_7 doublet and obtain the following periodic Anderson model

$$\mathcal{H}_{\text{PAM}} = \sum_{\mathbf{k},\mathbf{s}} \epsilon_{\mathbf{k},\mathbf{s}} c^{\dagger}_{\mathbf{k},\mathbf{s}} c_{\mathbf{k},\mathbf{s}} + \epsilon^{f}_{\Gamma_7} \sum_{i,\sigma} \tilde{f}^{\dagger}_{i,\sigma} \tilde{f}_{i,\sigma} + \sum_{\mathbf{k},\sigma,\mathbf{s}} \left(\tilde{V}_{\mathbf{k},\sigma\mathbf{s}} \tilde{f}^{\dagger}_{\mathbf{k},\sigma} c_{\mathbf{k},\mathbf{s}} + h.c. \right), \tag{S2}$$

where the constrained operators $\tilde{f}^{\dagger}_{i,\sigma}$ ($\tilde{f}_{i,\sigma}$) create (annihilate) an f-electron in the Γ_7 dou-

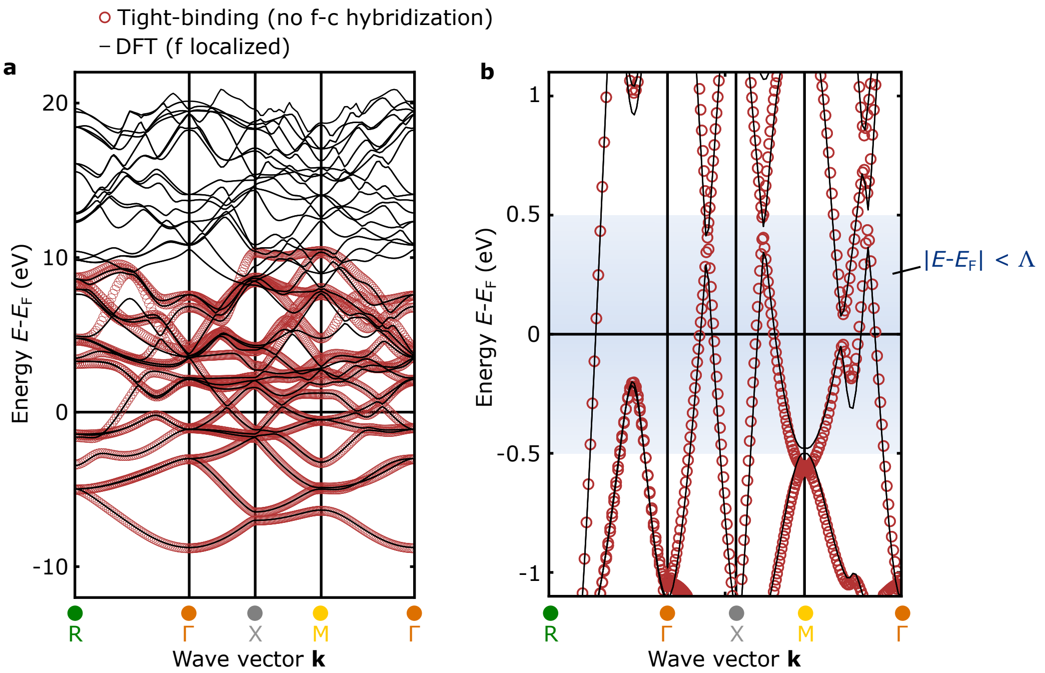

FIG. S2. **Electronic band structure of $CeIn_3$ with localized f electrons.** Comparison of the DFT calculated band structure with the f-electrons localized in the cores (solid lines) and the electronic structure of our 25 band tight-binding model with the hybridization between the f-orbitals (not shown) and the conduction bands (symbols) turned off. **a** Comparison including all the conduction bands. **b** Comparison near E_F, where the shaded region corresponds to the cutoff $\Lambda = 0.5\,\mathrm{eV}$ used in the calculation to separate the short- and long-range interactions.

blet with $\sigma = \{\uparrow, \downarrow\}$ and $\tilde{f}^{\dagger}_{i,\sigma}\tilde{f}^{\dagger}_{i,\sigma'} = 0$, $\epsilon^{f}_{\Gamma_7}$ is the energy of the Γ_7 states, and $\tilde{V}_{\mathbf{k},\sigma\mathbf{s}}$ is the hybridization between the Γ_7 doublet and the conduction electron states ($1 \leq s \leq 36$). s is a combined spin and orbital index for the conduction electrons.

By treating the small hybridization $\tilde{V}_{\mathbf{k},\sigma\mathbf{s}}$ as a perturbation, the periodic Anderson model can be reduced to an effective Kondo lattice model, which appears to second order in the hybridization, plus an effective exchange interaction between the Γ_7 doublets that is generated to fourth order in the hybridization, which is necessary to properly account for the magnetic interactions as detailed below:

$$\mathcal{H}_{\mathrm{PAM}} \to \mathcal{H}_{\mathrm{KLM}} + \mathcal{H}_{\mathrm{Heis}}. \tag{S3}$$

The Kondo lattice model Hamiltonian is

$$\mathcal{H}_{\mathrm{KLM}} = {\sum_{\mathbf{k},\mathbf{s}}}' \epsilon_{\mathbf{k},\mathbf{s}} c^{\dagger}_{\mathbf{k},\mathbf{s}} c_{\mathbf{k},\mathbf{s}} + {\sum_{\substack{i,\mathbf{k},\mathbf{k}', \\ \sigma,\sigma',s,s'}}}' J^{\mathbf{ks},\mathbf{k}'\mathbf{s}'}_{i,\sigma\sigma'} \tilde{f}^{\dagger}_{i,\sigma} \tilde{f}_{i,\sigma'} c^{\dagger}_{\mathbf{k}',\mathbf{s}'} c_{\mathbf{k},\mathbf{s}}, \tag{S4}$$

where the local Hilbert space is constrained by the condition $\sum_{\sigma} \tilde{f}^{\dagger}_{i,\sigma} \tilde{f}_{i,\sigma} = 1$, which implies that the f-electrons are localized in their orbitals and their effective spin σ is the only remaining f-degree of freedom. It is important to note that we have only retained the conduction electron states whose distance to the Fermi level is smaller than a cut-off Λ for both the hopping and the Kondo coupling terms (indicated in the constrained sums). The Kondo interaction is then given by

$$J^{\mathbf{ks},\mathbf{k}'\mathbf{s}'}_{i,\sigma\sigma'} \approx \frac{1}{N} \frac{\tilde{V}_{\mathbf{k},\sigma\mathbf{s}} \tilde{V}^{*}_{\mathbf{k}',\sigma'\mathbf{s}'}}{E_F - \epsilon^{f}_{\Gamma_7}} e^{i(\mathbf{k}-\mathbf{k}')\cdot\mathbf{r_i}}, \tag{S5}$$

where we have approximated the energy of the virtual processes $\epsilon_{\mathbf{k},\mathbf{s}} - \epsilon^{f}_{\Gamma_7}$ by $E_F - \epsilon^{f}_{\Gamma_7}$. The Heisenberg Hamiltonian ($\mathcal{H}_{\mathrm{Heis}}$) includes all magnetic interactions between the Γ_7 states generated through a fourth order degenerate perturbation theory of the periodic Anderson model Eq. (S2), which are not captured in the KLM of Eq. (S4). This includes all particle-particle (pp) processes at all energies (Fig. S3), which are not included in the Kondo model [5], and particle-hole (ph) processes that involve at least one virtual excited state outside the cut-off $|\epsilon_{\mathbf{k},\mathbf{s}} - E_F| \geq \Lambda$.

For strongly localized f-electrons, the magnetic Ruderman-Kittel-Kasuya-Yosida (RKKY) interaction dominates the Kondo effect. The Kondo lattice model can be further reduced to the RKKY Hamiltonian via second order degenerate perturbation theory in the Kondo interaction (i.e. fourth order in the hybridization $\tilde{V}_{\mathbf{k},\sigma\mathbf{s}}$). To properly account for all spin interactions from our MO-PAM that are up to fourth order in the hybridization we obtain a spin Hamiltonian as follows:

$$\mathcal{H}_{\mathrm{PAM}} \rightarrow \mathcal{H}_{\mathrm{spin}} = \mathcal{H}_{\mathrm{RKKY}} + \mathcal{H}_{\mathrm{Heis}} \tag{S6}$$

where

$$\mathcal{H}_{\mathrm{RKKY}} = \frac{1}{2} \sum_{i,j} \sum_{\sigma_1,\sigma'_1,\sigma_2,\sigma'_2} K^{\mathrm{RKKY}}_{\sigma_1\sigma'_1\sigma_2\sigma'_2}(i,j) \tilde{f}^{\dagger}_{i,\sigma_1} \tilde{f}_{i,\sigma'_1} \tilde{f}^{\dagger}_{j,\sigma_2} \tilde{f}_{j,\sigma'_2}, \tag{S7}$$

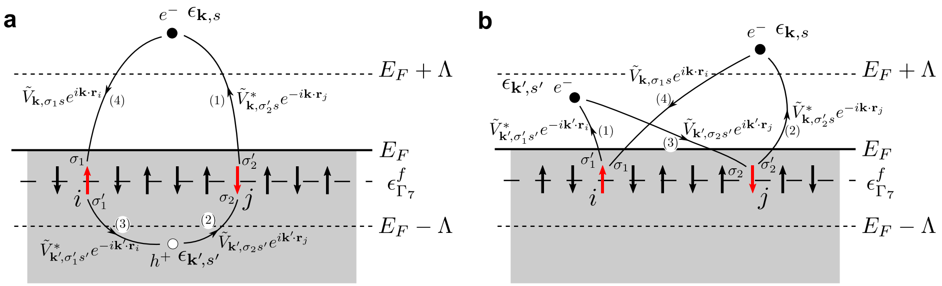

FIG. S3. Diagrams of the virtual processes $f^1 \to f^0 \to f^1$ in the particle-hole **a** and particle-particle **b** channels. Red and black short vertical arrows represent the spin (σ) of the localized electrons. The full (empty) dot represents a particle (hole) excitation with energy $\epsilon_{\boldsymbol{k},s}$ ($\epsilon_{\boldsymbol{k}',s'}$). The numbers (1) to (4) indicate the sequence of virtual processes that contribute to the effective exchange interaction between f-moments to fourth order in the hybridization. Each process is indicated with a black line with an arrow in the middle that is accompanied by the amplitude of the process. The variables σ_1 and σ_2 (σ_1' and σ_2') represent the initial (final) states of the f-moments i and j, respectively. The gray (white) region indicates the conduction band states below (above) the Fermi level. Dashed lines indicate the energy cut-off that restricts the excited states that contribute to the RKKY interactions (ph within the cut-off Λ), the superexchange (ph and pp involving at least one virtual state outside the cut-off), and a long-range interaction $\hat{K}^{(\mathrm{pp})}$ (pp within the cut-off).

and

$$\mathcal{H}_{\mathrm{Heis}} = \frac{1}{2} \sum_{i,j} \sum_{\sigma_1,\sigma_1',\sigma_2,\sigma_2'} K^{\mathrm{Heis}}_{\sigma_1\sigma_1'\sigma_2\sigma_2'}(i,j)\ \tilde{f}^{\dagger}_{i,\sigma_1} \tilde{f}_{i,\sigma_1'} \tilde{f}^{\dagger}_{j,\sigma_2} \tilde{f}_{j,\sigma_2'}. \tag{S8}$$

$\mathcal{H}_{\mathrm{spin}}$ can also be derived directly from the periodic Anderson model using fourth order perturbation theory. In doing so, $\mathcal{H}_{\mathrm{spin}}$ includes all the ph and pp fourth order processes in the hybridization $\tilde{V}_{\mathbf{k},\sigma\mathbf{s}}$, which are depicted in Fig. S3. The contributions from the ph channel, shown in Fig. S3**a**, produce an effective interaction

$$K^{(\mathrm{ph})}_{\sigma_1\sigma_1'\sigma_2\sigma_2'}(i,j) = \sum_{s,s'} \int_{\mathcal{B}} \frac{\mathrm{d}\mathbf{k}}{(2\pi)^3} \int_{\mathcal{B}} \frac{\mathrm{d}\mathbf{k'}}{(2\pi)^3} \frac{\tilde{V}^*_{\mathbf{k},\sigma_2' s} \tilde{V}_{\mathbf{k}',\sigma_2 s'} \tilde{V}^*_{\mathbf{k}',\sigma_1' s'} \tilde{V}_{\mathbf{k},\sigma_1 s}}{\epsilon_{\mathbf{k}',\mathbf{s}'} - \epsilon_{\mathbf{k},\mathbf{s}}} e^{i(\mathbf{k}-\mathbf{k}')\cdot(\mathbf{r}_i - \mathbf{r}_j)}$$
$$\times \left[\frac{f(\epsilon_{\mathbf{k}',\mathbf{s}'})[1 - f(\epsilon_{\mathbf{k},\mathbf{s}})]}{(\epsilon_{\mathbf{k},\mathbf{s}} - \epsilon^f_{\Gamma_7})^2} - \frac{f(\epsilon_{\mathbf{k},\mathbf{s}})[1 - f(\epsilon_{\mathbf{k}',\mathbf{s}'})]}{(\epsilon_{\mathbf{k}',\mathbf{s}'} - \epsilon^f_{\Gamma_7})^2} \right], \tag{S9}$$

where $\mathcal{B}$ stands for the first Brillouin zone. While the contributions from the pp channel,

shown in Fig. S3**b**, produce an effective coupling

$$K^{(\mathrm{pp})}_{\sigma_1\sigma_1'\sigma_2\sigma_2'}(i,j) = \sum_{s,s'} \int_{\mathcal{B}} \frac{\mathrm{d}\mathbf{k}}{(2\pi)^3} \int_{\mathcal{B}} \frac{\mathrm{d}\mathbf{k'}}{(2\pi)^3} \tilde{V}_{\mathbf{k},\sigma_1 s} \tilde{V}^*_{\mathbf{k'},\sigma_1' s'} \tilde{V}_{\mathbf{k'},\sigma_2 s'} \tilde{V}^*_{\mathbf{k},\sigma_2' s} \; e^{i(\mathbf{k}-\mathbf{k'})\cdot(\mathbf{r}_i-\mathbf{r}_j)} \\ \times \frac{\epsilon_{\mathbf{k},\mathbf{s}} + \epsilon_{\mathbf{k'},\mathbf{s'}} - 2\epsilon^f_{\Gamma_7}}{\left(\epsilon_{\mathbf{k'},\mathbf{s'}} - \epsilon^f_{\Gamma_7}\right)^2 \left(\epsilon_{\mathbf{k},\mathbf{s}} - \epsilon^f_{\Gamma_7}\right)^2} \; [1 - f(\epsilon_{\mathbf{k},\mathbf{s}})][1 - f(\epsilon_{\mathbf{k'},\mathbf{s'}})]. \tag{S10}$$

The Fourier transform of these effective interactions is:

$$K^{(\mathrm{ph})}_{\sigma_1\sigma_1'\sigma_2\sigma_2'}(\mathbf{q}) = \sum_{s,s'} \int_{\mathcal{B}} \frac{\mathrm{d}\mathbf{k}}{(2\pi)^3} \frac{\tilde{V}_{\mathbf{q}+\mathbf{k},\sigma_1 s} \tilde{V}^*_{\mathbf{k},\sigma_1' s'} \tilde{V}_{\mathbf{k},\sigma_2 s'} \tilde{V}^*_{\mathbf{q}+\mathbf{k},\sigma_2' s}}{\epsilon_{\mathbf{k},\mathbf{s'}} - \epsilon_{\mathbf{q}+\mathbf{k},\mathbf{s}}} \\ \times \left[\frac{f(\epsilon_{\mathbf{k},\mathbf{s'}})[1 - f(\epsilon_{\mathbf{q}+\mathbf{k},\mathbf{s}})]}{(\epsilon_{\mathbf{q}+\mathbf{k},\mathbf{s}} - \epsilon^f_{\Gamma_7})^2} - \frac{f(\epsilon_{\mathbf{q}+\mathbf{k},\mathbf{s}})[1 - f(\epsilon_{\mathbf{k},\mathbf{s'}})]}{(\epsilon_{\mathbf{k},\mathbf{s'}} - \epsilon^f_{\Gamma_7})^2} \right], \tag{S11}$$

$$K^{(\mathrm{pp})}_{\sigma_1\sigma_1'\sigma_2\sigma_2'}(\mathbf{q}) = \sum_{s,s'} \int_{\mathcal{B}} \frac{\mathrm{d}\mathbf{k}}{(2\pi)^3} \tilde{V}_{\mathbf{q}+\mathbf{k},\sigma_1 s} \tilde{V}^*_{\mathbf{k},\sigma_1' s'} \tilde{V}_{\mathbf{k},\sigma_2 s'} \tilde{V}^*_{\mathbf{q}+\mathbf{k},\sigma_2' s} \\ \times \frac{\epsilon_{\mathbf{q}+\mathbf{k},\mathbf{s}} + \epsilon_{\mathbf{k},\mathbf{s'}} - 2\epsilon^f_{\Gamma_7}}{\left(\epsilon_{\mathbf{k},\mathbf{s'}} - \epsilon^f_{\Gamma_7}\right)^2 \left(\epsilon_{\mathbf{q}+\mathbf{k},\mathbf{s}} - \epsilon^f_{\Gamma_7}\right)^2} \; [1 - f(\epsilon_{\mathbf{q}+\mathbf{k},\mathbf{s}})][1 - f(\epsilon_{\mathbf{k},\mathbf{s'}})]. \tag{S12}$$

In order to separate the contributions to $\mathcal{H}_{\mathrm{RKKY}}$ and $\mathcal{H}_{\mathrm{Heis}}$, we must split both interactions into two terms, $K^{(\mathrm{ph})}(\mathbf{q}) = \hat{\mathbf{K}}^{(\mathrm{ph})}(\mathbf{q}) + \check{\mathbf{K}}^{(\mathrm{ph})}(\mathbf{q})$ $[K^{(\mathrm{pp})}(\mathbf{q}) = \hat{\mathbf{K}}^{(\mathrm{pp})}(\mathbf{q}) + \check{\mathbf{K}}^{(\mathrm{pp})}(\mathbf{q})]$, where for $\hat{K}^{(\mathrm{ph})}(\mathbf{q})$ $[\hat{K}^{(\mathrm{pp})}(\mathbf{q})]$ the integration over momenta is restricted by the condition $|\epsilon_{\mathbf{k},\mathbf{s'}} - E_F| < \Lambda$ and $|\epsilon_{\mathbf{q}+\mathbf{k},\mathbf{s}} - E_F| < \Lambda$, with the cut-off $\Lambda = 0.5$ eV. The remaining contribution to the integral (at least one of the virtual states is outside the energy interval defined by the cut-off) is included in $\check{K}^{(\mathrm{ph})}(\mathbf{q})$ $[\check{K}^{(\mathrm{pp})}(\mathbf{q})]$. Now we can see that the RKKY contribution derived from the KLM [Eq. (S4)] only includes processes that contribute to $\hat{K}^{(\mathrm{ph})}(\mathbf{q})$. The remaining three terms ($\check{K}^{(\mathrm{ph})}(\mathbf{q})$, $\hat{K}^{(\mathrm{pp})}(\mathbf{q})$, and $\check{K}^{(\mathrm{pp})}(\mathbf{q})$) are interactions not accounted for in our KLM. Consequently, the sum of these three terms are defined as our Heisenberg contribution, and must be added to our low energy effective model Eq. (S3) if we aim to accurately account for magnetic interactions.

To derive the single-magnon dispersion, it is convenient to express the effective spin Hamiltonian in terms of the the pseudo spin-$\frac{1}{2}$ operators $\mathbf{S_i} \equiv \frac{\mathbf{1}}{\mathbf{2}}\tilde{\mathbf{f}}^\dagger_{\mathbf{i},\alpha}\sigma_{\alpha\beta}\tilde{\mathbf{f}}_{\mathbf{i},\beta}$:

$$\mathcal{H}_{\mathrm{spin}} = \frac{1}{2}\sum_{\mathbf{q},\nu} \tilde{I}^{\nu\nu}_{\mathbf{q}} S^\nu_{\mathbf{q}} S^\nu_{-\mathbf{q}} + \frac{1}{2}\sum_{\mathbf{q}} \left(\tilde{I}^{xy}_{\mathbf{q}} S^x_{\mathbf{q}} S^y_{-\mathbf{q}} + \tilde{I}^{yz}_{\mathbf{q}} S^y_{\mathbf{q}} S^z_{-\mathbf{q}} + \tilde{I}^{zx}_{\mathbf{q}} S^z_{\mathbf{q}} S^x_{-\mathbf{q}} \right), \tag{S13}$$

where

$$\tilde{I}^{xx}_{\mathbf{q}} = [K_{\uparrow\downarrow\downarrow\uparrow}(\mathbf{q}) + \mathbf{K}_{\downarrow\uparrow\uparrow\downarrow}(\mathbf{q}) + \mathbf{K}_{\uparrow\downarrow\uparrow\downarrow}(\mathbf{q}) + \mathbf{K}_{\downarrow\uparrow\downarrow\uparrow}(\mathbf{q})] \,, \tag{S14a}$$

$$\tilde{I}^{yy}_{\mathbf{q}} = [K_{\uparrow\downarrow\downarrow\uparrow}(\mathbf{q}) + \mathbf{K}_{\downarrow\uparrow\uparrow\downarrow}(\mathbf{q}) - \mathbf{K}_{\uparrow\downarrow\uparrow\downarrow}(\mathbf{q}) - \mathbf{K}_{\downarrow\uparrow\downarrow\uparrow}(\mathbf{q})] \,, \tag{S14b}$$

$$\tilde{I}^{zz}_{\mathbf{q}} = [K_{\uparrow\uparrow\uparrow\uparrow}(\mathbf{q}) + \mathbf{K}_{\downarrow\downarrow\downarrow\downarrow}(\mathbf{q}) - \mathbf{K}_{\uparrow\uparrow\downarrow\downarrow}(\mathbf{q}) - \mathbf{K}_{\downarrow\downarrow\uparrow\uparrow}(\mathbf{q})] \,, \tag{S14c}$$

$$\tilde{I}^{xy}_{\mathbf{q}} = 2i\,[-K_{\uparrow\downarrow\downarrow\uparrow}(\mathbf{q}) + \mathbf{K}_{\downarrow\uparrow\uparrow\downarrow}(\mathbf{q}) + \mathbf{K}_{\uparrow\downarrow\uparrow\downarrow}(\mathbf{q}) - \mathbf{K}_{\downarrow\uparrow\downarrow\uparrow}(\mathbf{q})] \,, \tag{S14d}$$

$$\tilde{I}^{yz}_{\mathbf{q}} = 2i\,[K_{\uparrow\downarrow\uparrow\uparrow}(\mathbf{q}) + \mathbf{K}_{\downarrow\uparrow\downarrow\downarrow}(\mathbf{q}) - \mathbf{K}_{\uparrow\downarrow\downarrow\downarrow}(\mathbf{q}) - \mathbf{K}_{\downarrow\uparrow\uparrow\uparrow}(\mathbf{q})] \,, \tag{S14e}$$

$$\tilde{I}^{zx}_{\mathbf{q}} = 2\,[K_{\uparrow\uparrow\uparrow\downarrow}(\mathbf{q}) - \mathbf{K}_{\downarrow\downarrow\downarrow\uparrow}(\mathbf{q}) + \mathbf{K}_{\uparrow\uparrow\downarrow\uparrow}(\mathbf{q}) - \mathbf{K}_{\downarrow\downarrow\uparrow\downarrow}(\mathbf{q})] \,, \tag{S14f}$$

with $K(\mathbf{q}) = \hat{\mathbf{K}}^{(\mathrm{ph})}(\mathbf{q}) + \check{\mathbf{K}}^{(\mathrm{ph})}(\mathbf{q}) + \hat{\mathbf{K}}^{(\mathrm{pp})}(\mathbf{q}) + \check{\mathbf{K}}^{(\mathrm{pp})}(\mathbf{q})$.

The effective spin-spin interaction turns out to be practically isotropic because of the absence of f^2 virtual states and the presence of the cubic symmetry:

$$\mathcal{H}_{\mathrm{spin}} = \mathcal{H}_{\mathrm{RKKY}} + \mathcal{H}_{\mathrm{Heis}} \simeq \frac{1}{2}\sum_{\mathbf{q},\nu} \tilde{I}_{\mathbf{q}} S^{\nu}_{\mathbf{q}} S^{\nu}_{-\mathbf{q}} \tag{S15}$$

with $\tilde{I}_{\mathbf{q}} = I^{\mathrm{RKKY}}_{\mathbf{q}} + I_{\mathbf{q}}$. Indeed, our numerical evaluation confirms that $\tilde{I}_{\mathbf{q}} \equiv \tilde{I}^{xx}_{\mathbf{q}} = \tilde{I}^{yy}_{\mathbf{q}} = \tilde{I}^{zz}_{\mathbf{q}}$. Furthermore, the terms $\{\tilde{I}^{xy}_{\mathbf{q}}, \tilde{I}^{yz}_{\mathbf{q}}, \tilde{I}^{zx}_{\mathbf{q}}\}$ that correspond to the cubic anisotropies are found to be much smaller than 0.01 meV.

The RKKY Hamiltonian is

$$\mathcal{H}_{\mathrm{RKKY}} = \frac{1}{2}\sum_{\mathbf{q},\nu} I^{\mathrm{RKKY}}_{\mathbf{q}} S^{\nu}_{\mathbf{q}} S^{\nu}_{-\mathbf{q}} \tag{S16}$$

where $I^{\mathrm{RKKY}}_{\mathbf{q}}$ has been derived from the Kondo lattice model. Due to the need to approximate the conduction electron particle hole states to lie at the Fermi energy, when deriving the Kondo lattice model, $I^{\mathrm{RKKY}}_{\mathbf{q}}$ is similar, but not exactly equal, to $\hat{I}^{(\mathrm{ph})}_{\mathbf{q}}$. A comparison shown in Fig. S4(a) illustrates the similarity, and hence, the validity in the approximation used to derive the Kondo lattice model. Fig. S4(b) shows a comparison between the magnon dispersions obtained including either $I^{\mathrm{RKKY}}_{\mathbf{q}}$ or $\hat{I}^{(\mathrm{ph})}_{\mathbf{q}}$ together with the Heisenberg contribution.

The Heisenberg term is

$$\mathcal{H}_{\mathrm{Heis}} = \frac{1}{2}\sum_{\mathbf{q},\nu} I_{\mathbf{q}} S^{\nu}_{\mathbf{q}} S^{\nu}_{-\mathbf{q}} \tag{S17}$$

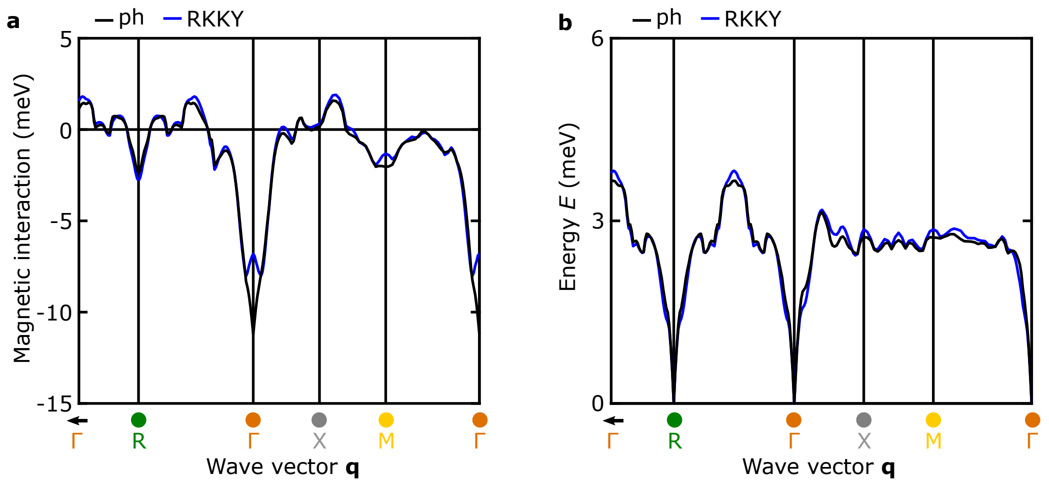

FIG. S4. (a) Comparison between RKKY interactions $\hat{I}^{(\mathrm{ph})}_{\mathbf{q}}$ (black line) and $I^{\mathrm{RKKY}}_{\mathbf{q}}$ (blue line) derived from the PAM and from the KLM, respectively. (b) Shown is the magnon dispersion on the path RΓXMΓ obtained directly from the PAM, i.e., using $\hat{I}^{(\mathrm{ph})}_{\mathbf{q}}$ (black line), as well as the dispersion resulting from the approximated RKKY interaction $I^{\mathrm{RKKY}}_{\mathbf{q}}$ (blue line) that is obtained from the KLM. The same Heisenberg contribution is included for both magnon dispersions shown in (b).

with $I_{\mathbf{q}} \equiv I^{\mathrm{SE}}_{\mathbf{q}} + \hat{I}^{(\mathrm{pp})}_{\mathbf{q}}$. The effective superexchange interaction $I^{\mathrm{SE}}_{\mathbf{q}} \equiv \check{I}^{(\mathrm{ph})}_{\mathbf{q}} + \check{I}^{(\mathrm{pp})}_{\mathbf{q}}$ involves at least one virtual excited state outside the energy interval defined by the cut-off Λ.

In the calculation, the position of the Fermi level, $E_F = 12.588$ eV, is fixed by the number of non-f electrons per unit cell. The only free parameter of the theory is the diagonal energy $\epsilon^{f}_{\Gamma_7}$ of the Γ_7 doublet. By fitting the bandwidth of the measured single-magnon dispersion we obtain $\epsilon^{f}_{\Gamma_7} \approx 12.076$ eV for the effective spin-spin interaction derived from the PAM (using $\hat{I}^{(\mathrm{ph})}_{\mathbf{q}}$) and $\epsilon^{f}_{\Gamma_7} \approx 12.009$ eV for the effective spin-spin interaction derived from the KLM (using $I^{\mathrm{RKKY}}_{\mathbf{q}}$), which is the case presented in the main text.

II. EXPERIMENTAL METHODS

In the following, we elaborate on the experimental methods. After a description of the sample preparation, details of the neutron-spectroscopy experiments are presented.

A. Sample preparation

Figure S5 shows a photograph of the $CeIn_3$ sample-mosaic on the aluminum holder. The mosaic spread with respect to the $(\bar{1}10)$ rotation axis of the single-crystal pieces around the ideal orientation, which is indicated schematically on the right, is within ± 1.4 deg rotation angle. Shown in **b** is an examplary Laue picture that indicates the high quality of one of the single-crystal pieces.

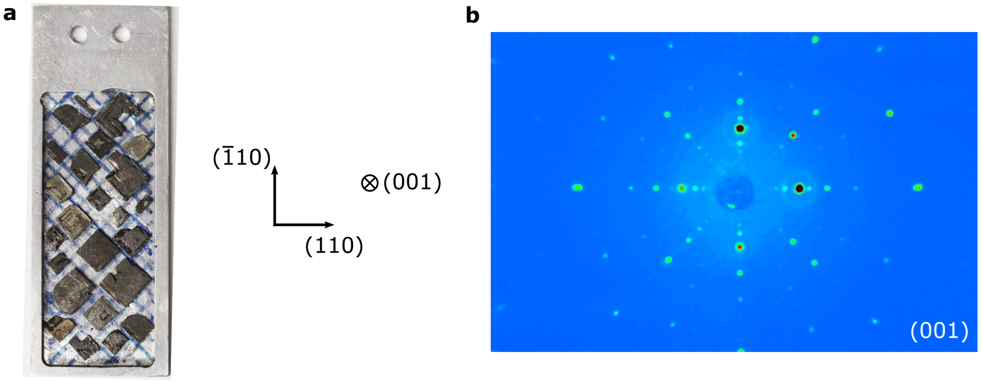

FIG. S5. **a** Photograph of the $CeIn_3$ single-crystal mosaic. The crystallographic directions along that the crystals were coaligned are indicated on the right. **b** X-ray Laue diffraction pattern of one of the used $CeIn_3$ single-crystal samples illustrating the high quality. The data were recorded with the X-ray beam parallel to the (001) crystallographic direction.

B. Neutron spectroscopy experiments

To determine the dispersion of magnetic excitations in $CeIn_3$ at $T = 1.8\,\mathrm{K}$, inelastic neutron scattering (INS) experiments were performed using the time-of-flight technique at the cold neutron chopper spectrometer (CNCS) at Oak Ridge National Laboratory (ORNL) [6]. The orientation of the $CeIn_3$ sample mosaic for the experiment was such that the crystallographic direction $(1\bar{1}0)$ was vertical and (110) as well as (001) were in the horizontal scattering plane of CNCS (see Fig. S4).

The CNCS spectrometer was operated in the following two complementary instrumental settings:

- In the first setting, which we refer to as high-energy setting, the incident neutron energy was $E_{i,1} = 12\,\mathrm{meV}$ (wave vector $K_{i,1} = 2.406\,\text{Å}^{-1}$) and the choppers of CNCS were operated in the high-flux mode with 180 Hz double-disk rotation frequency.

- In the second setting, which we refer to as high-resolution setting, the incident neutron energy was $E_{i,2} = 3.315\,\mathrm{meV}$ (wave vector $K_{i,2} = 1.265\,\text{Å}^{-1}$) and the choppers were operated in the high-flux mode with 300 Hz double-disk rotation frequency.

The first setting provides an overview of the magnetic excitations in a large area of reciprocal space and up to high energy transfers, whereas the second setting permits high resolution measurements of the magnon dispersion in the vicinity of the momentum transfer $\mathbf{Q_0} = \left(-\frac{\mathbf{1}}{\mathbf{2}}, -\frac{\mathbf{1}}{\mathbf{2}}, \frac{\mathbf{1}}{\mathbf{2}}\right)$, which corresponds to probing excitations at the magnetic propagation vector $\mathbf{q}_{\mathrm{AFM}} = \left(\frac{\mathbf{1}}{\mathbf{2}}, \frac{\mathbf{1}}{\mathbf{2}}, \frac{\mathbf{1}}{\mathbf{2}}\right)$ [7] located at the R point.

For the data sets in the high-energy and high-resolution setting, respectively, the angular coverage of sample rotation amounted to 33 deg and to 50 deg with a step size of 1 deg.

Figure S6 illustrates the areas of reciprocal space that were covered in the high-energy setting (cf. **a**) and in the high-resolution setting (cf. **b**) showing maps of elastic neutron-scattering intensity in the horizontal scattering plane.

The pronounced Gaussian profile of the magnetic Bragg peak at $(-0.5, -0.5, 0.5)$, as recorded in the high-resolution setting, indicates an excellent sample mosaicity with respect to the $(\bar{1}10)$ axis.

INS-data were recorded at 1.8 K (foreground) and at 20 K (background), i.e., in the magnetically ordered state and well above the ordering temperature. At each temperature, a horace scan was performed, i.e., the sample was rotated around the vertical axis. Data reduction was performed with the software *HORACE* [8]. The size of the background-corrected magnetic intensity, $I(\mathbf{Q}, \omega)$, reflects the dynamic magnetic scattering cross section, which is given by:

$$\mathrm{d}^2\sigma/\mathrm{d}\Omega\mathrm{d}\omega = \frac{N}{\hbar}\frac{K_f}{K_i}\left(\frac{g\gamma r_0}{2}\right)^2 \exp\left(-2W\right) F^2(\mathbf{Q}) \sum_{\alpha,\beta}\left(\delta_{\alpha\beta} - \hat{\mathbf{Q}}_\alpha\hat{\mathbf{Q}}_\beta\right)\mathbf{S}^{\alpha\beta}(\mathbf{Q},\omega)\,, \qquad \text{(S18)}$$

where $S^{\alpha\beta}$ denotes thy dynamic magnetic spin-correlation function, $\exp(-2W)$ denotes the

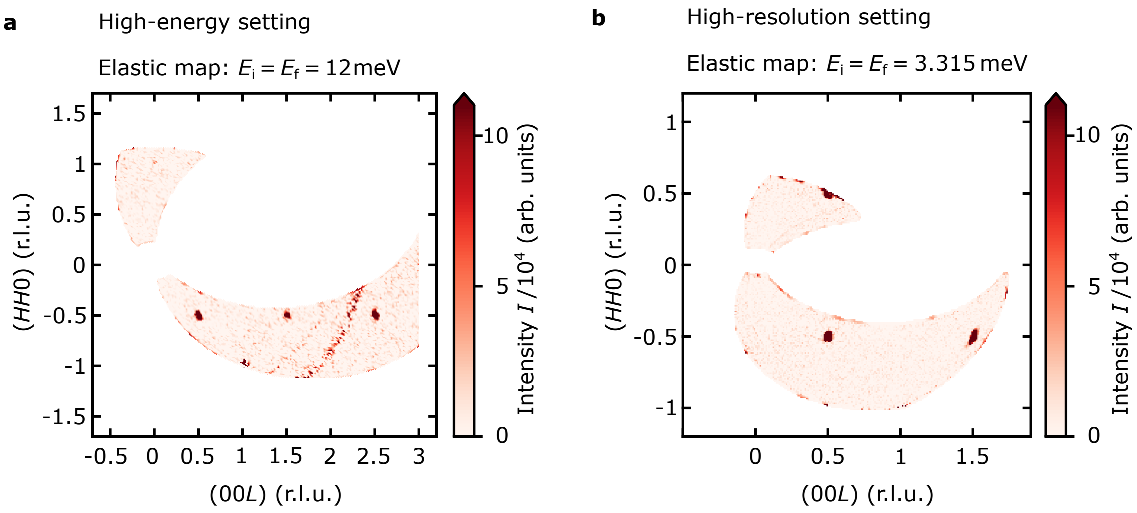

FIG. S6. **Maps of elastic neutron scattering intensity recorded in the horizontal scattering plane. a** In the high-energy setting a large area of reciprocal space is accessible. Clearly visible are magnetic Bragg peaks at the momentum transfers, $\mathbf{Q} = (-0.5, -0.5, 0.5)$, $(-0.5, -0.5, 1.5)$ and $(-0.5, -0.5, 2.5)$ as well as a relatively weak temperature dependent signal at the structural positions $(1, 1, 0)$ and $(-1, -1, 1)$. **b** In the high-resolution setting the accessible range in reciprocal space is restricted to the close vicinity around the magnetic Bragg peak position $(-0.5, -0.5, 0.5)$. Both panels show background-subtracted neutron scattering intensity that was integrated over the energy range $\Delta E = \pm 0.1\,\mathrm{meV}$.

Debye-Waller factor, F the magnetic form factor, N the number of unit cells, and $\frac{\gamma r_0}{2}$ the magnetic scattering length.

The dynamic magnetic correlation function is directly related to the imaginary part of the dynamic magnetic susceptibility, $\chi^{\alpha\beta}(\mathbf{Q}, \omega)$, by the fluctuation-dissipation theorem:

$$\chi^{\alpha\beta\prime\prime}(\mathbf{Q}, \omega) = \mathbf{g^2}\mu_\mathrm{B}^\mathbf{2}\frac{\pi}{\hbar}\left(\mathbf{1} - \exp\left(-\frac{\hbar\omega}{\mathbf{k}_\mathrm{B}\mathbf{T}}\right)\right)\mathbf{S}^{\alpha\beta}(\mathbf{Q}, \omega) \tag{S19}$$

Detailed accounts one these relations are found, e.g., in Refs. [9, 10]. An introduction into the theory of magnetic neutron scattering is further provided by Ref. [11].

Setting $\tilde{\chi}''(\mathbf{Q}, \omega) := \sum_{\alpha,\beta}\left(\delta_{\alpha\beta} - \hat{\mathbf{Q}}_\alpha\hat{\mathbf{Q}}_\beta\right)\chi^{\alpha\beta\prime\prime}(\mathbf{Q}, \omega)$ we find further [9–11]:

$$\tilde{S}(\mathbf{Q}, \omega) = \frac{\mathbf{13.77b^{-1}\tilde{I}(Q}, \omega)}{\mathbf{g^2F^2}\exp\left(-\mathbf{2W}\right)\mathbf{Nk_fR_0}} \tag{S20}$$

where $\tilde{I}(\mathbf{Q}, \omega)$ denotes the time-of-flight scattering intensity.

To present scattering intensity on an absolute scale, we followed the procedure explained in Ref. [10], normalizing the spectroscopy data to the incoherent scattering of the sample,

$I_{ic} = \int \tilde{I}(\mathbf{Q}, \omega)\mathrm{d}\mathbf{E}$ using the relation:

$$N k_f R_0 = 4\pi \frac{I_{ic}}{\sum_j \sigma^{ic} e^{-2W_j}} \, . \tag{S21}$$

The experimentally observed incoherent scattering contains on the one hand contributions due to the sample, which are dominated by the scattering off indium having the cross section $\sigma^{ic} = 0.54 \cdot b$ [12]. On the other hand, incoherent scattering arises from the aluminium sample holder. We assumed that the entire $CeIn_3$ sample and all aluminum in the sample holder contributed to the observed incoherent scattering. To assess the uncertainty in this normalization procedure, we assumed that we may have overestimated the amount of aluminum in the beam by 70 %. In addition, we considered that the procedure introduced in Ref. [10] is generally expected to suffer uncertainties of the order of 20 % on the resulting values due to systematic errors.

The resolution volume R_0 as a function of energy was calculated by the *Violini*-method using the software *Takin* (see Refs. [13, 14]) for the high-energy setting in the entire energy-range and for the high-resolution setting below 1.4 meV energy transfers. For the high-resolution measurements above 1.4 meV energy transfer we assumed a constant resolution, as the *Violini*-method failed.

The neutron spectroscopy data were further corrected for absorption effects. Using a ray-tracing technique, a comprehensive sample-geometry dependent absorption correction due to indium, which in our case dominates the absorption, was performed. The required code for the absorption correction is available at `https://github.com/wolfgangsimeth/NeutronAbsorptionPlatelikeSamples`. Here the angle and energy dependent path of neutrons passing through the sample was considered in order to determine the intensity reduction due to the absorption. For this the samples shown in Fig. S5 were estimated to be perfectly plate-like with an average thickness 0.78 mm. In the entire relevant parameter range, incident neutrons hit the platelets at one of their large facets, scatter, and exit the platelet on the opposite site (see Fig. S7). The absorption cross sections on the paths l_1 and l_2 depend on the wave-lengths before and after scattering. The tabulated value for indium in Ref. [12], $\sigma^a = 193.8 \cdot b$, refers to neutrons of wave-length 1.798 $\mathring{A}$. We assumed a linear dependence of σ^a on the incident neutron wavelength λ. As a further constraint we limited the absorption coefficient to 0.1 in order to avoid divergences arising from specific constella-

tions, where the scattered beam is parallel to the sample surface. These points corresponded to momentum and energy transfers that are far away from the settings in that the magnon dispersions in Fig. 3 and Fig. 4 of the main text was recorded.

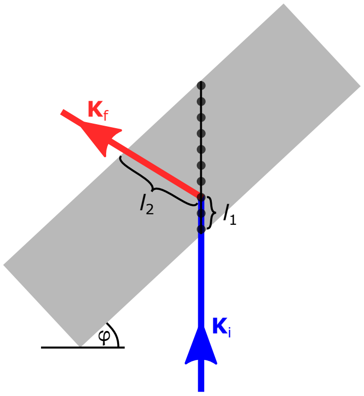

FIG. S7. **Schematic illustration of the absorption correction.** The single-crystal mosaic comprises of plate-like samples for that the in-plane dimensions are much larger than their thickness. For a given incident wave-vector $\mathbf{K_i}$ (blue arrow), each momentum and energy transfer corresponds to a unique combination of scattered wave-vector $\mathbf{K_f}$ (red arrow) and rotation angle ϕ of the sample. For each such combination, the path of unscattered neutrons through the sample (gray rectangle), as indicated by a black thin line, was divided into 10 pieces (black circles) that scatter neutrons with equal probability. We determined the average absorption for the neutron paths l_1 and l_2 within the sample.

To compare experiments with the dynamic spin correlation function $S^{\alpha.\beta}(\boldsymbol{Q}, \omega)$ from MO-PAM calculations, we calculated the dynamic magnetic susceptibility for $g = \frac{6}{7}$. A comparison between experiment and theory on absolute scale is illustrated in Fig. 3 of the main text. The agreement is excellent, and even reproduces fine details in the structure of the intensity distribution (see. Fig. 3 (c) and (d) in the main text), in particular, when the size of the systematic errors in the conversion to absolute units (see above) as well as uncertainties in the calculation of the dynamic susceptibility are taken into account. Notably, for the calculation of the dynamic magnetic susceptibility, we note that additional effects such as frustration, Kondo screening, or quantum fluctuations can reduce the magnetic moment and the resulting dynamic magnetic susceptibility substantially. In the related material $CeRhIn_5$, we found that these effects can be about 20 % [15].

III. MAGNON DISPERSION INFERRED FROM EXPERIMENTAL DATA

In the following, we present a detailed account of the magnetic excitations in $CeIn_3$ as derived from our INS experiments. At first, we show neutron spectroscopy data that are complementary to the data in the main text. Subsequently, the excitations at the R point are studied in detail and the presence of a putative gap, previously reported by Knafo *et al.* [16], is discussed. Finally, the trajectory of the magnetic dispersion throughout the Brillouin zone is inferred from our data.

A. Complementary INS data

Figure S8 presents neutron spectroscopy data of magnetic excitations on the path RM and over several Brillouin zones.

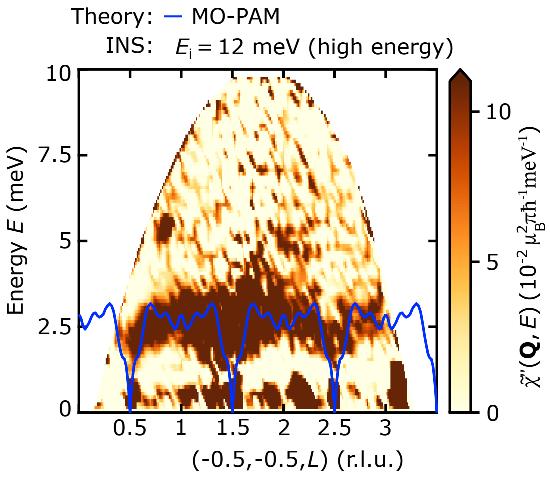

FIG. S8. **Magnon dispersion on the path RM.** The imaginary part of the dynamic susceptibility, $\tilde{\chi}''(\mathbf{Q}, \mathbf{E})$, inferred from our experiments (see text for details) is illustrated. Intensity was integrated over a Q-range of distance 0.173 r.l.u. along the direction $(1\bar{1}0)$. The blue solid line denotes the magnon dispersion calculated via the MO-PAM.

B. Magnetic excitations at the R point

The INS data recorded in the high-resolution setting reveal a relatively steep slope of the magnon dispersion emerging from the magnetic Bragg peak at $\mathbf{q}_{\mathrm{AFM}} = \left(\frac{1}{2}, \frac{1}{2}, \frac{1}{2}\right)$, as presented in Fig. 4 of the main text. As elaborated on in the following, the data indicate further, that a gap at the R point is either absent or smaller than the energy resolution of

CNCS. In particular, the data pose an upper bound on the size of a putative gap at the R point.

The instrumental energy resolution may be inferred from the incoherent scattering intensity at a **Q**-position where elastic magnetic Bragg scattering is absent. The energy-dependent foreground intensity at $\mathbf{Q_1} = \left(-\frac{1}{2}, -\frac{1}{2}, \frac{1}{2} - \mathbf{0.1}\right)$, which is obtained by integration of time-of-flight intensity over a cuboid **Q**-volume centered at $\mathbf{Q_1}$ and extended over a distance of ± 0.018 r.l.u. along the directions (110), $(1\bar{1}0)$, and (001) displays a Gaussian profile around the elastic line given by:

$$G(E) = I_0 \cdot \exp\left(-\frac{(E - E_0)^2}{2\sigma^2}\right) . \tag{S22}$$

The width of the profile, which is characterised by $\sigma = 45\,\mu\text{eV}$ and a full width at half maximum $\text{FWHM} = 2\sqrt{2\ln 2}\sigma = 106\,\mu\text{eV}$, essentially represents the resolution of CNCS in the high-resolution setting at zero energy transfer. The value is close to resolution of CNCS reported in literature (cf. Ref. [6, 17]) and considerably smaller than $196\,\mu\text{eV}$, which is the FWHM for incoherent Vanadium scattering estimated by the *Violini*-algorithm by means of the software *Takin* (see Ref. [14]).

The R point was investigated at the momentum transfer $\mathbf{Q_0} = \left(-\frac{1}{2}, -\frac{1}{2}, \frac{1}{2}\right)$. Figure S9**a** presents background-subtracted INS data as a function of energy transfer E at the position $\mathbf{Q_0}$, where the elastic magnetic Bragg peak is located, as well as at the shifted position $\mathbf{Q_1}$, where magnetic intensity is absent. The magnetic Bragg peak at $\mathbf{Q_0}$ as a function of energy, E, displays a Gaussian profile with the same width as the instrumental resolution. The magnon dispersion intersects due to its steep slope with the integration dome of $\mathbf{Q_0}$ up to energies around 1 meV (see Fig. S9**b**).

The onset of substantial scattering intensity at energy transfers right above the elastic Bragg peak indicates that the dispersion is gapless within the energy resolution of CNCS.

C. Trajectory of Magnon Dispersion

The trajectory in reciprocal space of the magnon dispersion was inferred from INS data from the high-energy and high-resolution setting, as explained in the following.

Sufficiently far away from the points R and Γ and in major parts of the Brillouin zone, the

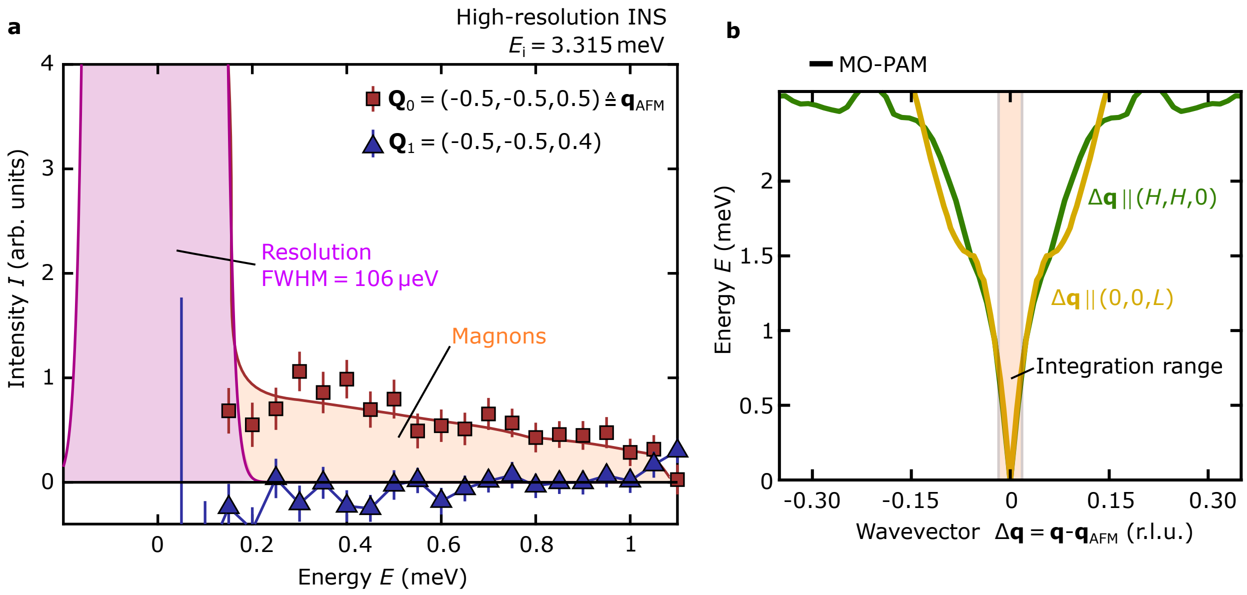

FIG. S9. **Gapless magnon dispersion with extremely high slope at the** R **point. a** INS intensity at the reciprocal space position $\mathbf{Q_0}$ (brown square symbols connected by a guide for the eyes), which corresponds to the R point, indicates finite intersection with the magnon dispersion up to energies around 1 meV. The resulting neutron intensity is indicated by orange shading. In contrast, the scattering intensity at $\mathbf{Q_1}$ (blue triangle symbols connected by lines), which is slightly shifted from the R point, is vanishingly small in the entire energy-transfer range. The plotted intensity was obtained by integrating over a $\mathbf{Q}$-volume extended over a distance of ± 0.018 r.l.u. along the directions (110), (1$\bar{1}$0), and (001). Error bars denote the statistical error. The Gaussian profile with pink shading corresponds to the energy resolution of the instrument. **b** Schematic view of the experimental integration range at $\mathbf{Q_0}$ compared to the magnon dispersion as inferred from MO-PAM calculations. The solid lines correspond to the calculated magnon dispersion along the cubic face diagonal (green) and the cubic edge (yellow). The integration range around $\mathbf{Q_0}$ is indicated by the brown shading.

magnetic dispersion is relatively flat and the trajectory is clearly visible in constant $\mathbf{Q}$-cuts through the INS data that were recorded in the high-energy setting.

Figure S10 presents high-energy INS-intensity as a function of energy at several $\mathbf{Q}$-positions on the paths RΓ, ΓX, XM, and MΓ. The peaks that are visible indicate the intercepts with the magnon dispersion and were fitted with Gaussian profiles. Their centers indicate the location of the dispersion trajectory.

Close to the R point, where the magnetic ordering vector is located, the gapless magnetic excitation dispersion displays a steep slope and the trajectory is visible in cuts at constant energy transfers through the high-resolution INS data set. Figs. S11, S12, and S13 present constant-energy cuts for momentum transfers around $\mathbf{Q_0} = \left(-\frac{1}{2}, -\frac{1}{2}, \frac{1}{2}\right)$. For the cuts at finite energies, i.e., $E > 0$, the data were corrected by the Bose-factor and the resulting

intensity represents the imaginary part of the susceptibility at constant E.

As a function of $\Delta\mathbf{q} = \mathbf{q} - \mathbf{q}_{\mathrm{AFM}}$ along the three reciprocal-space directions $\left(\bar{H}\bar{H}H\right)$, $\left(\bar{H}\bar{H}0\right)$, and $(00L)$ the magnon dispersion features two intersections in constant-energy cuts taken at energies $\leq 2.2\,\mathrm{meV}$. Due to the reciprocity of spin waves the intersections are symmetric around the center $\mathbf{q}_{\mathrm{AFM}}$, i.e., they appear at values $+\mathbf{q}$ and $-\mathbf{q}$, but possibly with different spectral weights.

At the lowest energies, the separation of intersection points is smaller than the FWHM of the experimental momentum-transfer-resolution (purple shading) and the cuts are well fitted by a single-Gaussian profile (orange shading) denoted $1G$. For higher energies the splitting exceeds the FWHM of the instrumental resolution, i.e., $2\delta > f_{\mathrm{res}}$, and the profile can better be fitted by two Gaussian peaks being symmetric around the center.

The width as a function of $\mathbf{Q}$ of the magnetic Bragg peak at momentum $\mathbf{Q_0} = \left(-\frac{1}{2}, -\frac{1}{2}, \frac{1}{2}\right)$ (zero energy transfer) essentially represents the instrumental resolution convoluted with the mosaicity of the invesitgated sample, which for the directions $\left(\bar{H}\bar{H}H\right)$, $\left(\bar{H}\bar{H}0\right)$, and $(00L)$ is described by a Gaussian profile with FWHM of $f_{(\bar{H}\bar{H}H)} \approx 0.054\,\mathrm{\mathring{A}}^{-1}$, $f_{(HH0)} \approx 0.063\,\mathrm{\mathring{A}}^{-1}$, and $f_{(00L)} \approx 0.035\,\mathrm{\mathring{A}}^{-1}$, respectively. The values indicate an excellent mosaicity of the sample array that is smaller than $\pm 1.4\,\mathrm{deg}$ around the vertical axis. The ratio for the instrumental resolution along $(\bar{H}\bar{H}H)$ and $(00L)$ is in agreement with the values inferred with the *Violini*-algorithm by means of the *Takin* software (see Ref. [14]), where $f^{\mathrm{Violini}}_{(\bar{H}\bar{H}H)} \approx 0.027\,\mathrm{\mathring{A}}^{-1}$ and $f^{\mathrm{Violini}}_{(00L)} \approx 0.015\,\mathrm{\mathring{A}}^{-1}$ were obtained.

Experimental data points of magnon trajectories that are presented in Figs. 3 and 4 of the manuscript were inferred from the Gaussian fit profiles that were presented in this section and the errorbars of data points represent one σ of the fitted Gaussian profiles.

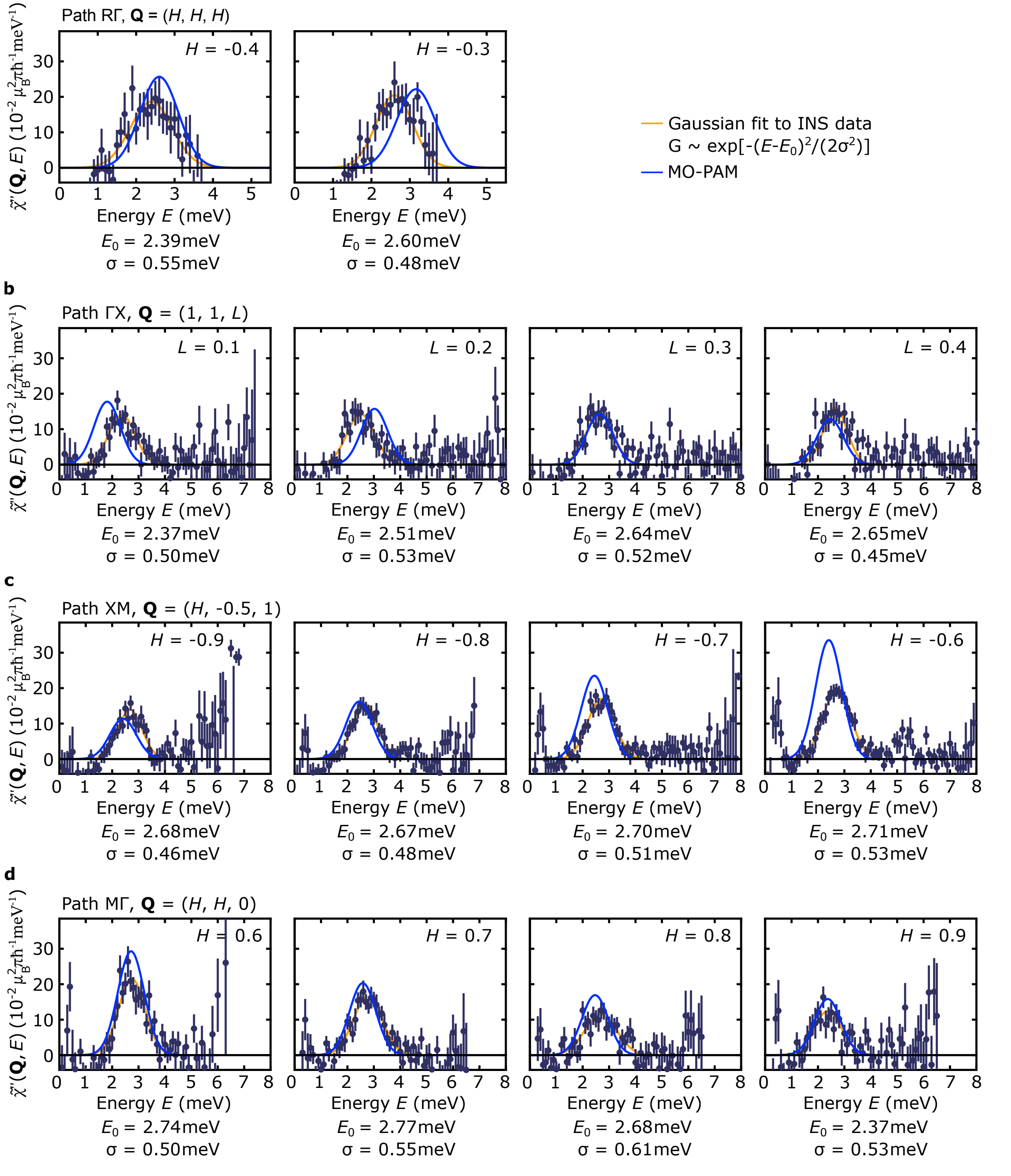

FIG. S10. **Energy cuts through $\tilde{\chi}''(\mathbf{Q},\mathbf{E})$, as inferred from the INS data set recorded in the high-energy setting, at constant Q-positions distributed over the entire Brillouin zone.** **a**, **b**, **c**, and **d** present cuts at **Q**-positions that lie on the paths RΓ, ΓX, XM, and MΓ, respectively. The intersections with the magnon dispersion are characterised by Gaussian profiles (center E_0, FWHM $2\sqrt{2\ln 2}\sigma$). Each data point on the path $\Gamma XM\Gamma$ represents intensity that was integrated over a distance of ±0.17 r.l.u. along three perpendicular **Q**-directions. Error bars denote the statistical error. On the path $R\Gamma$ the integration ranges were ±0.09 r.l.u. and ±0.17 r.l.u. for $(\bar{1}\bar{1}1)$ and the other axes, respectively. On MΓ the integration range was ±0.10 r.l.u. and ±0.17 r.l.u. for $(\bar{1}\bar{1}0)$ and the perpendicular axes, respectively. The solid orange and blue lines represent a Gaussian fitted to the data and the intensity distribution as calculated by the MO-PAM, respectively.

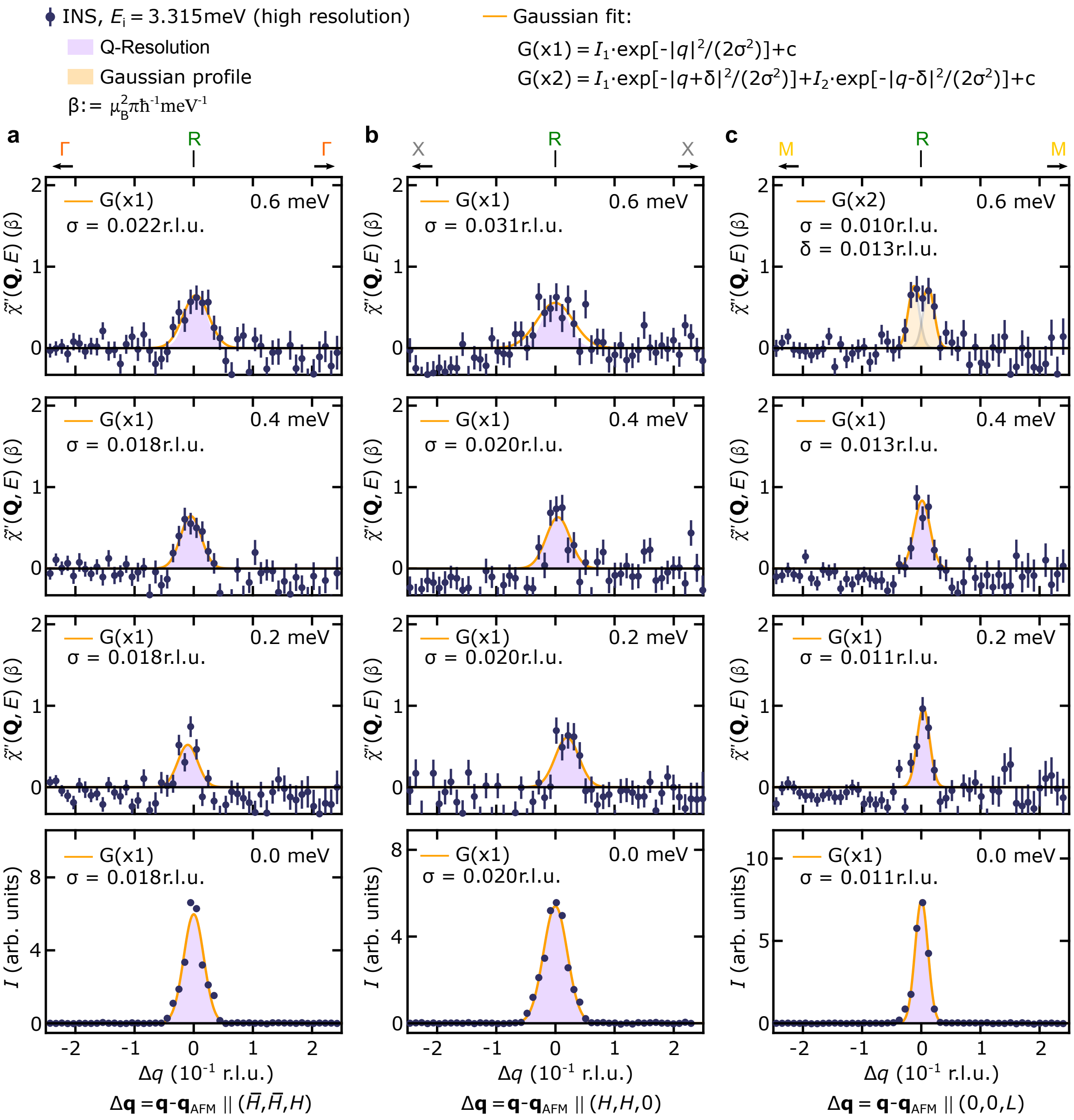

FIG. S11. **Magnetic excitations in the vicinity of the antiferromagnetic ordering vector at fixed energies** ($0\,\mathrm{meV} \leq E \leq 0.6\,\mathrm{meV}$)**.** Shown are cuts through high-resolution INS-data at constant energies as a function of Δq, whereby $\mathbf{q} = \mathbf{q} - \mathbf{q}_{\mathrm{AFM}}$ is directed along the **a** cubic space diagonal RΓ, **b** cubic face diagonal RX, and **c** cubic edge RM, respectively. Error bars denote the statistical error. The data points that are shown were recorded at momentum transfers around $\mathbf{Q} = (\mathbf{-0.5}, \mathbf{-0.5}, \mathbf{0.5})$.

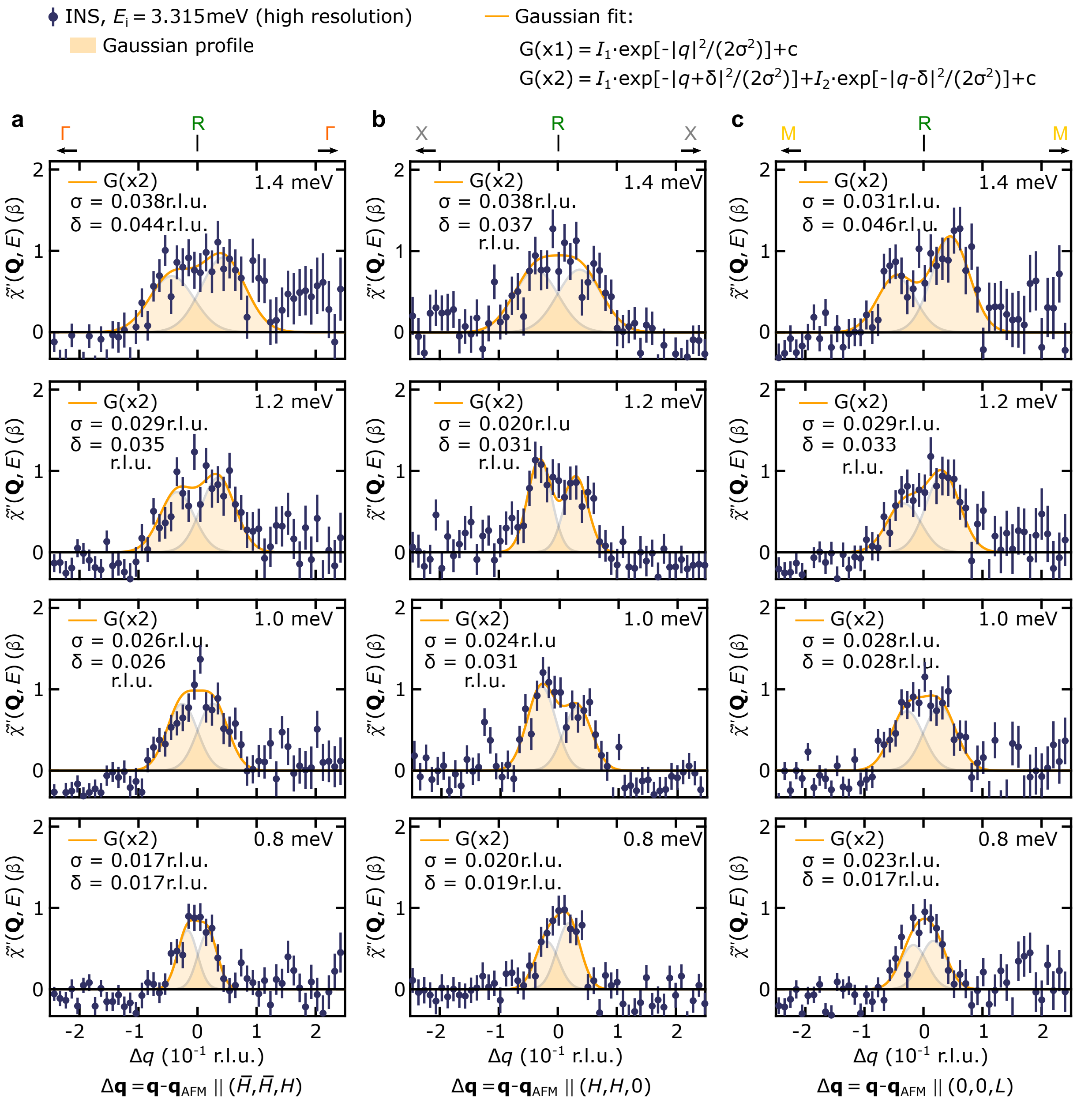

FIG. S12. **Magnetic excitations in the vicinity of the antiferromagnetic ordering vector at fixed energies ($0.8\,\mathrm{meV} \leq E \leq 1.4\,\mathrm{meV}$).** Shown are cuts through high-resolution INS-data at constant energies as a function of Δq, whereby $\mathbf{q} = \mathbf{q} - \mathbf{q}_{\mathrm{AFM}}$ is directed along the **a** cubic space diagonal RΓ, **b** cubic face diagonal RX, and **c** cubic edge RM, respectively. Error bars denote the statistical error. The data points that are shown were recorded at momentum transfers around $\mathbf{Q} = (-\mathbf{0.5}, -\mathbf{0.5}, \mathbf{0.5})$.

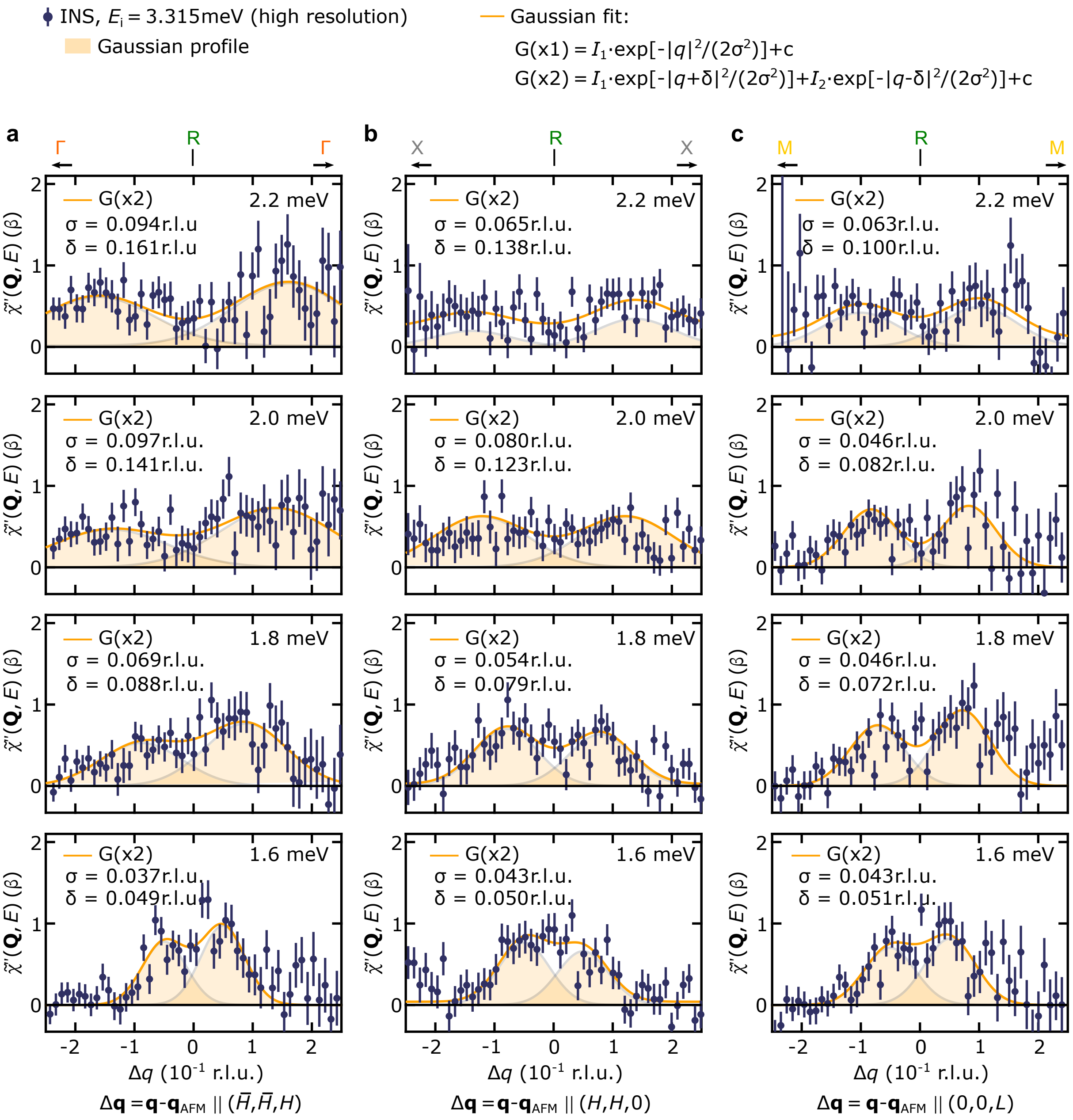

FIG. S13. **Magnetic excitations in the vicinity of the antiferromagnetic ordering vector at fixed energies** ($1.6\,\text{meV} \leq E \leq 2.2\,\text{meV}$)**.** Shown are cuts through high-resolution INS-data at constant energies as a function of Δq, whereby $\mathbf{q} = \mathbf{q} - \mathbf{q}_{\text{AFM}}$ is directed along the **a** cubic space diagonal RΓ, **b** cubic face diagonal RX, and **c** cubic edge RM, respectively. Error bars denote the statistical error. The data points that are shown were recorded at momentum transfers around $\mathbf{Q} = (\mathbf{-0.5}, \mathbf{-0.5}, \mathbf{0.5})$..

IV. MAGNON VELOCITY

The magnon group velocity at ordering vector $\mathbf{q}_{\mathrm{AFM}} = \left(\frac{1}{2}, \frac{1}{2}, \frac{1}{2}\right)$ along a direction $\Delta\mathbf{q} = \mathbf{q} - \mathbf{q}_{\mathrm{AFM}}$ is given by:

$$v_{\Delta\mathbf{q}} = \hat{e}_{\Delta\mathbf{q}} \cdot \nabla E_{\mathbf{k}}\big|_{\mathbf{k}=\mathbf{q}_{\mathrm{AFM}}}, \tag{S23}$$

where $\hat{e}_{\Delta\mathbf{q}} \equiv \Delta\mathbf{q}/|\Delta\mathbf{q}|$.

Magnon velocities at the R point, i.e., $\mathbf{q}_{\mathrm{AFM}} = \left(\frac{1}{2}, \frac{1}{2}, \frac{1}{2}\right)$, were determined by linear fits to high-resolution INS data with momentum around $\mathbf{Q_0} = \left(-\frac{1}{2}, -\frac{1}{2}, \frac{1}{2}\right)$ and compared with computations.

A. Magnon velocities inferred from experimental data

Magnon velocities were determined for the three cases, where $\Delta\mathbf{q}$ is directed along the cubic space diagonal RΓ, the cubic face diagonal RX, and the cubic edge RM. The dispersions along the three directions are presented in Fig. 4 of the manuscript. At small wave vectors $\Delta\mathbf{q}$ with respect to the R point and energy transfers $\leq 1.6\,\mathrm{meV}$ the magnetic excitations display essentially a linear dispersion.

At lowest energies, where the distance of the two intersection points in constant-energy cuts as a function of $\Delta q = |\mathbf{q} - \mathbf{q}_{\mathrm{AFM}}|$ (see Figs. S11, S12, and S13) is smaller than the FWHM of the instrumental resolution, an estimate for the magnon velocity is obtained from the resolution. For the cubic space diagonal RΓ, the splitting is smaller than the experimental resolution for energies $E \leq 0.6\,\mathrm{meV}$, which imposes a conservative lower bound on the magnon velocity:

$$v_{\mathrm{R}\Gamma} > \frac{0.6\,\mathrm{meV}}{\frac{1}{2} \cdot f_{(\bar{H}\bar{H}H)}} \approx 30 \cdot \frac{\mathrm{meV}}{\mathrm{r.l.u.}}, \tag{S24}$$

where we used $1\,\mathrm{r.l.u.} \approx 1.340\,\mathring{\mathrm{A}}^{-1}$ for $\mathrm{CeIn_3}$ at ambient pressure.

We assume a gapless magnon dispersion and determine the magnon velocities by linear weighted least-squares fits to the data points inferred from constant-energy cuts at energies $E = 0.8\,\mathrm{meV}$, $1\,\mathrm{meV}$, .., $1.6\,\mathrm{meV}$. We performed four fits, where we considered the $N = 2$,

TABLE S1. **Magnon velocities at the R point as inferred from experimental data. Shown are the values along the cubic space diagonal RΓ, the cubic face diagonal RX, and the cubic edge RM**. The magnon velocity was determined by least square fits considering several sets of sample points that are described in the text. Uncertainties correspond to statistical errors.

Number of sampling points:	**Magnon velocities** $v_{\Delta\mathbf{q}}$ (meV/ r.l.u.)		
N	$\Delta\mathbf{q}$ ‖RΓ	$\Delta\mathbf{q}$ ‖RX	$\Delta\mathbf{q}$ ‖RM
2	42.2 ± 4.2	35.5 ± 4.3	47.0 ± 0.1
3	38.6 ± 3.3	37.3 ± 2.5	43.8 ± 3.8
4	36.7 ± 2.8	37.4 ± 1.9	41.5 ± 3.2
5	34.6 ± 1.9	36.1 ± 1.8	37.7 ± 3.4
6			36.5 ± 2.9
mean±std	38.0 ± 1.6	36.6 ± 0.5	41.3 ± 1.9

3, ..., 5 data points with lowest energies and inferred the mean values. For the cubic face diagonal, the same energies $E = 0.8\,\text{meV}$, $1\,\text{meV}$, .., $1.6\,\text{meV}$ were considered and fits were carried out for the $N = 2$, 3, ..., 5 data points with lowest energies. In turn, for the cubic edge, constant-energy cuts at $E = 0.6\,\text{meV}$, $0.8\,\text{meV}$, .., $1.6\,\text{meV}$ were considered and fits were carried out for the $N = 2$, 3, ..., 6 data points with lowest energies.

The magnon velocities obtained from the fits as well as the mean values for the three reciprocal space directions are presented in Tab. S1. The velocity averaged over the three directions RΓ, RX, and RM is given by $v_{\Delta\mathbf{q}} = (38.6 \pm 0.8)$ meV/ r.l.u. .

B. Magnon velocities inferred from MO-PAM calculations

The magnon dispersion inferred from MO-PAM calculations displays essentially linear behavior at energies between 0 meV and 1 meV for the three reciprocal-space directions RΓ, RX, and RM. The slopes for the three directions as inferred from weighted least squares fits with a linear function passing through the origin result in the velocities $v_{\mathrm{R\Gamma}} = (43.5 \pm 1.5)$ meV/ r.l.u., $v_{\mathrm{RX}} = (45.2 \pm 1.7)$ meV/ r.l.u., and $v_{\mathrm{RM}} = (47.4 \pm 1.2)$ meV/ r.l.u.. The velocity averaged over the three directions RΓ, RX, and RM is given by $v_{\Delta\mathbf{q}} = (45.3{\pm}0.9)$ meV/ r.l.u..

V. BANDWIDTH

The bandwidth of magnetic excitations was determined on the dispersion on the subpath ΓXMΓ (note, that the subpath RΓ is not fully covered by INS data). The dispersion obtained

via our experiment has the bandwidth $W_{\rm exp} = (2.75 \pm 0.03)$ meV, whereby the maximum of the recorded dispersion is attained on the subpath MΓ. The dispersion inferred from MO-PAM calculations has a slightly larger bandwith given by $W_{\rm MO-PAM} = (3.18 \pm 0.02)$ meV and the corresponding maximum is attained on the subpath ΓX.

The size of the bandwidth is well reproduced by fits with Heisenberg models. The bandwidths of the J_1 fit, J_1-J_2 fit, J_1-J_2-J_3 fit, and J_1-J_2-J_3-J_4 fit on the path RΓXMΓ are given by 2.91 meV, 3.24 meV, 3.02 meV, and 3.11 meV, respectively. Here J_ν denotes the Heisenberg exchange connecting the νth nearest neighbor pair.

VI. RATIO OF MAGNON VELOCITY AND BANDWIDTH

The steep dispersion at the R point manifests itself in the extremely large ratio of magnon velocity to bandwidth, which is typically expressed in dimensionless units. The ratio as inferred from experimental data amounts to:

$$\eta_{\rm exp} = \left(\frac{v_{\Delta\mathbf{q}}}{W}\right)_{\rm exp} \cdot \frac{1 \cdot \text{r.l.u.}}{2\pi} = 2.23 \pm 0.06. \tag{S25}$$

Calculations that are based on MO-PAM result in a similarly large value given by:

$$\eta_{\text{MO-PAM}} = \left(\frac{v_{\Delta\mathbf{q}}}{W}\right)_{\text{MO-PAM}} \cdot \frac{1 \cdot \text{r.l.u.}}{2\pi} = 2.27 \pm 0.05. \tag{S26}$$

In contrast, finite series expansions using exchange constants up to a few nearest-neighbors typically feature substantially smaller values. The corresponding fits are shown in section VII. The J_1 fit, J_1-J_2 fit, J_1-J_2-J_3 fit, and J_1-J_2-J_3-J_4 fit lead to $\eta = \frac{1}{\sqrt{3}} \approx 0.58$, $\eta = 0.56$, $\eta = 0.90$, and $\eta = 0.91$ (for the ratio of magnon velocity at R in direction RΓ to bandwidth on the path RΓXMΓ), respectively.

VII. FIT OF EXPERIMENTAL DATA BY HEISENBERG MODELS

The magnon dispersion on the path RΓXMΓ, which is presented in Fig. 3 of the main text, was fitted with low-order nearest neighbour Heisenberg models. To reproduce the

bandwidth of measured magnons, the models were fitted by means of least-squares statistics to the 14 data-points that were recorded in the high-energy setting.

The excitation dispersion of a Heisenberg model can be expressed in terms of:

$$E\left(\mathbf{q}\right) = \frac{1}{2}\sqrt{\left|\left(\gamma_{\mathbf{q}_{\mathrm{AFM}}} - \gamma_{\mathbf{q}}\right)\cdot\left(\gamma_{\mathbf{q}_{\mathrm{AFM}}} - \gamma_{\mathbf{q}_{\mathrm{AFM}}+\mathbf{q}}\right)\right|}, \tag{S27}$$

with $\mathbf{q}_{\mathrm{AFM}} = (\mathbf{\frac{1}{2}}, \mathbf{\frac{1}{2}}, \mathbf{\frac{1}{2}})$ and $\mathbf{q} = (\mathbf{q_1}, \mathbf{q_2}, \mathbf{q_3})$. Accounting for exchange constants up to fourth nearest neighbours, $\gamma_{\mathbf{q}}$ is given by:

$$\begin{aligned}
\gamma_{\mathbf{q}} = {} & 2J_1\left[\cos(2\pi q_1) + \cos(2\pi q_2) + \cos(2\pi q_3)\right] \\
& + 2J_2\left[\cos(2\pi(q_1+q_2)) + \cos(2\pi(q_1-q_2)) + \cos(2\pi(q_3+q_1))\right. \\
& \qquad \left. + \cos(2\pi(q_3-q_1)) + \cos(2\pi(q_2+q_3)) + \cos(2\pi(q_2-q_3))\right] \\
& + 2J_3\left[\cos(2\pi(q_1+q_2+q_3)) + \cos(2\pi(-q_1+q_2+q_3))\right. \\
& \qquad \left. + \cos(2\pi(q_1-q_2+q_3)) + \cos(2\pi(q_1+q_2-q_3))\right] \\
& + 2J_4\left[\cos(4\pi q_1) + \cos(4\pi q_2) + \cos(4\pi q_3)\right].
\end{aligned} \tag{S28}$$

The J_1-fit (for which we set $J_2 = J_3 = J_4 = 0$) resulted in $J_1 = 0.97$, the J_1-J_2-fit ($J_3 = J_4 = 0$) in $J_1 = 1.34$ and $J_2 = 0.13$, the J_1-J_2-J_3-fit ($J_4 = 0$) in $J_1 = 0.38$, $J_2 = 0.04$, and $J_3 = 0.52$, and the J_1-J_2-J_3-J_4-fit in $J_1 = 0.22$, $J_2 = 0.00$, $J_3 = 0.69$, and $J_4 = 0.16$ (in units meV). Figure S14 shows the four Heisenberg fits in comparison with experimental data. The bandwidth that was recorded by INS is reproduced by each of the fits. In contrast, the magnon-velocity at the R point, as inferred by a linear fit to INS data, is distinctively larger than the slope of each of the Heisenberg fits.

VIII. CURRAT-AXE SPURIONS AND STEEP MAGNON DISPERSIONS

We now illustrate why triple-axis spectroscopy is not well-suited to investigate the steep dispersion that we observe in $CeIn_3$. This is due to spurious scattering that is caused by neutrons scattered incoherently at the monochromator or analyzer crystals of a triple-axis spectrometer. When the spectrometer is set to a non-zero energy transfer, i.e., $|\mathbf{K_i}| \neq |\mathbf{K_f}|$, but the angle between $\mathbf{K_i}$ and $\mathbf{K_f}$ as well as the orientation of the sample correspond to a geometry that allows for Bragg scattering from the sample, the incoherently scattered

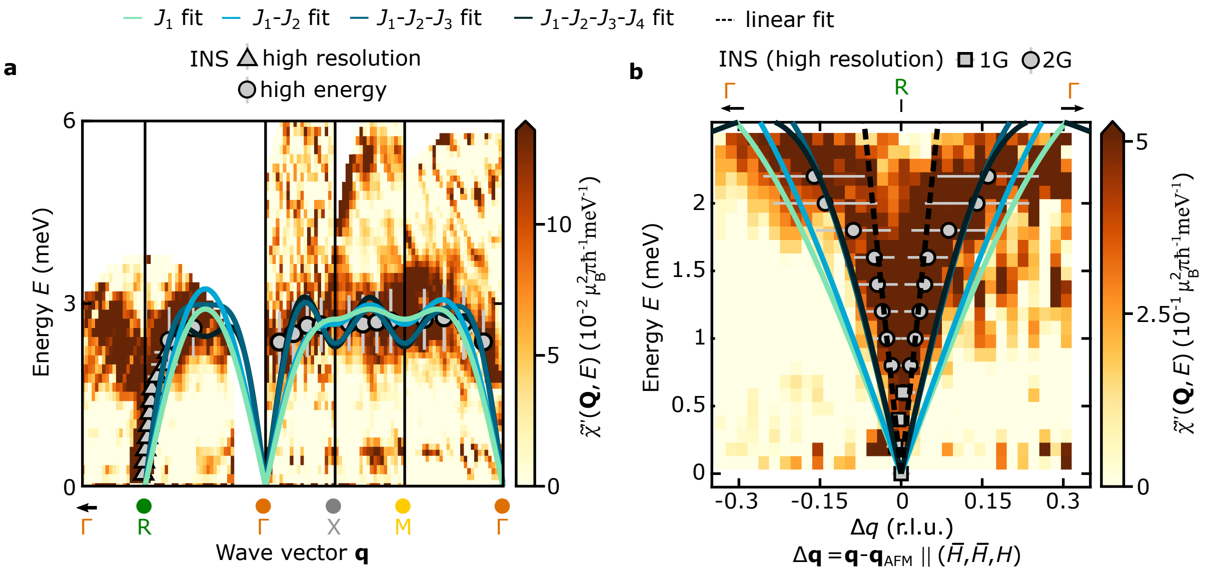

FIG. S14. **Fit of high-energy experimental data by Heisenberg models.** The neutron scattering data obtained via the high-energy setting were fitted by a J_1 Heisenberg-model, J_1-J_2 model, J_1-J_2-J_3 model, and J_1-J_2-J_3-J_4 model, respectively, as explained in the text. **a** Comparison of these fits (solid lines) with data of the high-energy setting (color-coding). The triangle and circle symbols correspond to maxima of Gaussian fits to cuts through the high-resolution and high-energy INS data, respectively. Error bars denote the standard deviations of these profiles. **b** Comparison of the fits (solid lines) with data of the high-resolution setting (color-coding). The square and circle symbols correspond to maxima of single and double Gaussian profiles, respectively, fitted to cuts through the high-resolution INS data. Error bars denote the standard deviations of these profiles. The doted lines correspond to linear fits to the INS data.

neutrons from the monochromator that have a wave vector $|\mathbf{K}_{\mathbf{i},\mathrm{inc}}| = |\mathbf{K}_{\mathbf{f}}|$ lead to the observation of accidental Bragg scattering. These scattering events, which may also alternatively arise from neutrons incoherently scattered at the analyzer, result in spurious intensity that appears as a dispersive feature close to a magnetic zone center with a linear dispersion. In the literature, this is well-known as Currat-Axe spurion [18].

To show this, we have repeated our experiment on $CeIn_3$ previously carried out on CNCS at ORNL, using the multiplexing triple-axis spectrometer CAMEA [19]. For each of the three incident neutron energies, spectroscopy data were recorded with two different positions of the multiplexing detector, namely $2\theta = -45$ deg and -41 deg, and with different angles of vertical sample rotation covering an angular range of 60 deg with a step size of 0.5 deg.

The branches of Currat-Axe spurions originating from the monochromator as well as from the analyzer were calculated by means of the software *MJOLNIR* [20] and are shown in Fig. S15. Note that these lines disperse in close vicinity of the real magnon dispersion,

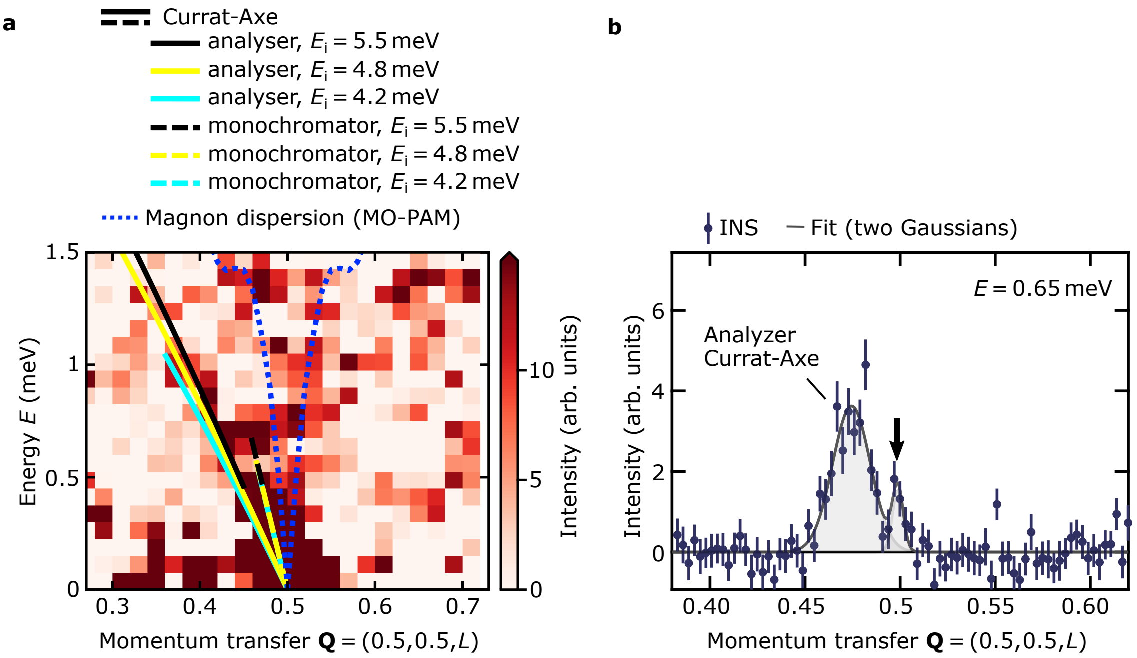

FIG. S15. **Triple axis spectroscopy and Currat-Axe spurions** Shown in **a** is neutron spectroscopy intensity around momentum transfers (0.5, 0.5, 0.5), which represents the R-point. Background recorded at 20 K were subtracted from the foreground data recorded at 2 K. INS intensity was integrated within a reciprocal space distance of ±0.09 r.l.u. along the axis $(1\bar{1}0)$. The location of Currat-Axe spurions from the analyzer crystal and from the monochromator are indicated by solid and interrupted lines, respectively. The spurions were calculated for incident neutron energies 4.2 meV (cyan color), 4.8 meV (yellow color), and 5.5 meV (black), respectively. The blue dotted line shows the trajectory of the magnon dispersion resulting from MO-PAM calculations. Shown in **b** is an exemplary **Q**-cut at constant energy 0.65 meV. INS intensity was integrated over a reciprocal space distance ±0.035 r.l.u. parallel to the axis $(1\bar{1}0)$ and over energies between 0.5 meV and 0.8 meV. The error bars denote the statistical error. The binning for the (001) direction amounts to 0.02 r.l.u.. The data were fitted with a superposition of two Gaussian profiles. The peak at smaller L arises from spurious scattering from the analyzer, whereas the peak in the center (black arrow) may be a superposition of spurious scattering from the monochromator and of magnons.

that is, however, substantially weaker. In the constant-energy cut shown in **b** the Currat-Axe spurions from incoherent analyzer scattering result in a peak at around $L = 0.47$ r.l.u. (Gaussian at smaller L). The signal in the center (Gaussian at larger L indicated by a black arrow) may be superposition of magnon scattering as well as Currat-Axe spurions arising from incoherent scattering at the monochromator.

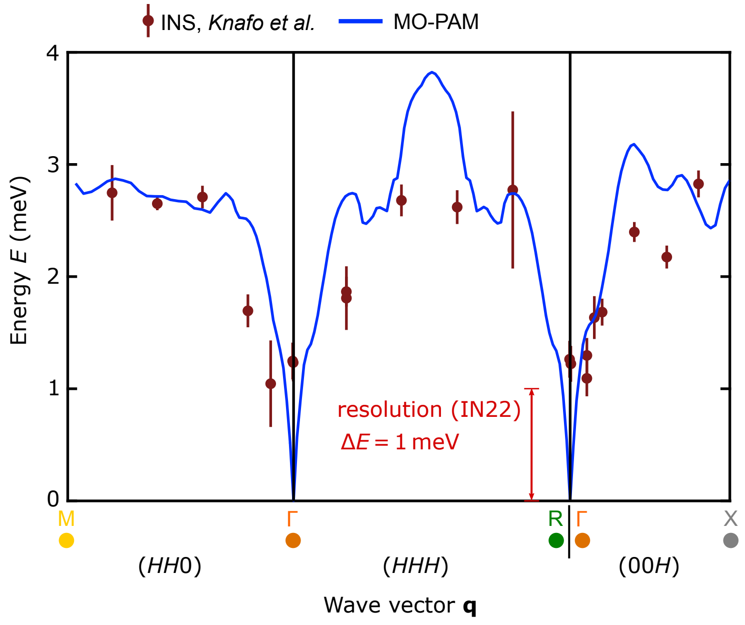

FIG. S16. **Comparison of MO-PAM calculations with previous INS-data.** Experimental data that were measured at IN22 and reported by *Knafo et al.* [16] are compared with MO-PAM calculations from this study. The red arrow indicates the size of the instrumental resolution.

IX. PREVIOUS EXPERIMENTAL STUDIES OF MAGNETIC EXCITATIONS

The magnetic excitations in $CeIn_3$ were previously characterised by *Knafo et al.* [16] using triple-axis spectroscopy. The data, which are presented in Fig. S16, are qualitatively in agreement with our study in major parts of the Brillouin zone. In particular, the recorded dispersion features the same bandwidth that we identified in our study. In the vicinity of Γ and in the vicinity of R, the energy resolution of IN22 was not sufficient to resolve the extremely steep excitation dispersion.

The authors of Ref. [16] fitted the dispersion of excitations with a J_1-J_2 model, which we identify in our report as inadequate to account for the excitations in $CeIn_3$ (see Sec. VII). In particular, the J_1-J_2 model is unable to reproduce the relatively large ratio of magnon velocity to bandwith, which we observed. In addition, the authors of Ref. [16] inferred a gap of size 1.28 meV at the magnetic zone center, i.e., at the R point, which is in stark contrast to our results indicating a gapless dispersion at the magnetic zone center.

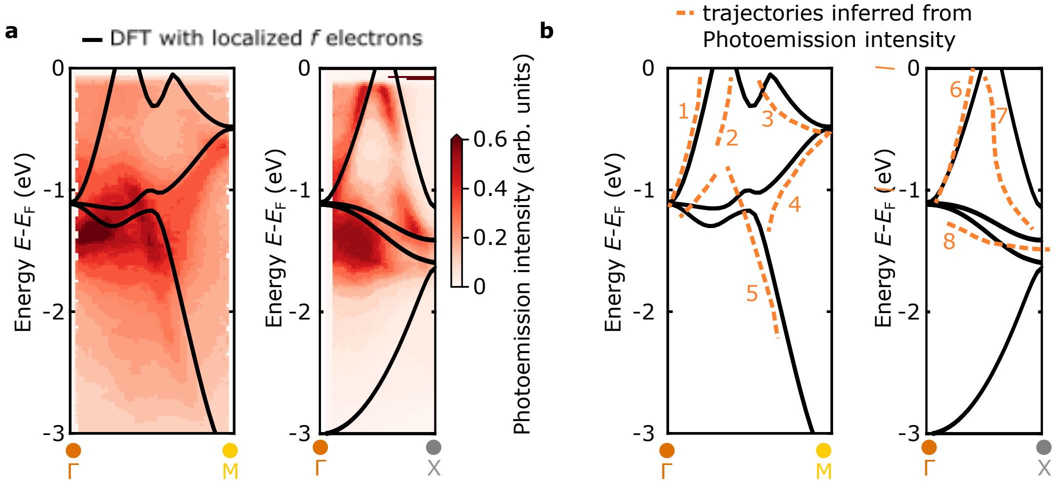

FIG. S17. **Comparison of DFT-calculations with angle-resolved photo emsission spectroscopy (ARPES) studies.** **a** Polarization dependent valence band structure obtained by ARPES measurements, as reported in Ref. [21] and provided in terms of the color-coding, as compared to the band structure from our DFT calculations obtained for localised f-electrons (black lines). The ARPES data were extracted from Fig. 2 of Ref. [21]. **b** Trajectories of the band structure inferred from local maxima of the ARPES data (orange dotted lines) compared with the trajectories obtained from our DFT calculations (black lines).

X. COMPARISON OF DFT CALCULATIONS WITH THE BAND STRUCTURE OBTAINED BY PHOTO EMISSION STUDIES

Fig. S17 presents a comparison of the DFT band structure shown in Fig. S2 with the polarization dependent valence band structure obtained by angle-resolved photo emission spectroscopy (ARPES) measurements (cf. Ref. [21]). Considering the challenges that arise when comparing surface sensitive ARPES data to DFT calculations as well as difficulties in ARPES experiments on cubic materials, the agreement between DFT and ARPES is excellent. Notably, in cubic materials the momentum resolution along the surface normal can be broadened resulting in difficulties of interpreting the ARPES data [22].

[1] P. Giannozzi, O. Andreussi, T. Brumme, O. Bunau, M. B. Nardelli, M. Calandra, R. Car, C. Cavazzoni, D. Ceresoli, M. Cococcioni, N. Colonna, I. Carnimeo, A. D. Corso, S. de Gironcoli, P. Delugas, R. A. DiStasio, A. Ferretti, A. Floris, G. Fratesi, G. Fugallo, R. Gebauer, U. Gerstmann, F. Giustino, T. Gorni, J. Jia, M. Kawamura, H.-Y. Ko, A. Kokalj, E. Küçükbenli, M. Lazzeri, M. Marsili, N. Marzari, F. Mauri, N. L. Nguyen, H.-V. Nguyen, A. O. de-la Roza, L. Paulatto, S. Poncé, D. Rocca, R. Sabatini, B. Santra, M. Schlipf, A. P. Seitsonen, A. Smogunov, I. Timrov, T. Thonhauser, P. Umari, N. Vast, X. Wu, and S. Baroni, J. Phys.: Condens. Matter **29**, 465901 (2017).

[2] W. Dijkman, W. De Groot, F. De Boer, and P. De Chatel, Valence Instabilities, Proceedings of the International Conference , 547 (1982).

[3] A. Dal Corso, Computational Materials Science **95**, 337 (2014).

[4] G. Pizzi, V. Vitale, R. Arita, S. Blügel, F. Freimuth, G. Géranton, M. Gibertini, D. Gresch, C. Johnson, T. Koretsune, J. Ibañez-Azpiroz, H. Lee, J.-M. Lihm, D. Marchand, A. Marrazzo, Y. Mokrousov, J. I. Mustafa, Y. Nohara, Y. Nomura, L. Paulatto, S. Poncé, T. Ponweiser, J. Qiao, F. Thöle, S. S. Tsirkin, M. Wierzbowska, N. Marzari, D. Vanderbilt, I. Souza, A. A. Mostofi, and J. R. Yates, J. Phys.: Condens. Matter **32**, 165902 (2020).

[5] P. Fazekas, *Lecture Notes on Electron Correlation and Magnetism* (WORLD SCIENTIFIC, 1999).

[6] G. Ehlers, A. A. Podlesnyak, J. L. Niedziela, E. B. Iverson, and P. E. Sokol, Rev. Sci. Instrum. **82**, 085108 (2011).

[7] J. M. Lawrence and S. M. Shapiro, Phys. Rev. B **22**, 4379 (1980).

[8] R. A. Ewings, A. Buts, M. D. Le, J. van Duijn, I. Bustinduy, and T. G. Perring, Nuclear Instruments and Methods in Physics Research Section A: Accelerators, Spectrometers, Detectors and Associated Equipment **834**, 132 (2016).

[9] M. Janoschek, P. Das, B. Chakrabarti, D. L. Abernathy, M. D. Lumsden, J. M. Lawrence, J. D. Thompson, G. H. Lander, J. N. Mitchell, S. Richmond, M. Ramos, F. Trouw, J.-X. Zhu, K. Haule, G. Kotliar, and E. D. Bauer, Sci. Adv. (2015), 10.1126/sciadv.1500188.

[10] G. Xu, Z. Xu, and J. M. Tranquada, Rev. Sci. Instrum. **84**, 083906 (2013).

[11] S. Lovesey, *Theory of Neutron Scattering from Condensed Matter*, Vol. 2 (Clarendon Press, 1984).

[12] V. F. Sears, Neutron News **3**, 26 (1992).

[13] N. Violini, J. Voigt, S. Pasini, and T. Brückel, Nuclear Instruments and Methods in Physics Research Section A: Accelerators, Spectrometers, Detectors and Associated Equipment **736**, 31 (2014).

[14] T. Weber, R. Georgii, and P. Böni, SoftwareX **5**, 121 (2016).

[15] D. M. Fobes, E. D. Bauer, J. D. Thompson, A. Sazonov, V. Hutanu, S. Zhang, F. Ronning, and M. Janoschek, Journal of Physics: Condensed Matter **29**, 17LT01 (2017).

[16] W. Knafo, S. Raymond, B. F. k, G. Lapertot, P. C. Canfield, and J. Flouquet, J. Phys.: Condens. Matter **15**, 3741 (2003).

[17] M. Anastasopoulos, R. Bebb, K. Berry, J. Birch, T. Bryś, J.-C. Buffet, J.-F. Clergeau, P. P. Deen, G. Ehlers, P. van Esch, S. M. Everett, B. Guerard, R. Hall-Wilton, K. Herwig, L. Hultman, C. Höglund, I. Iruretagoiena, F. Issa, J. Jensen, A. Khaplanov, O. Kirstein, I. L. Higuera, F. Piscitelli, L. Robinson, S. Schmidt, and I. Stefanescu, J. Inst. **12**, P04030 (2017).

[18] G. Shirane, S. M. Shapiro, and J. M. Tranquada, *Neutron Scattering with a Triple-Axis Spectrometer: Basic Techniques* (Cambridge University Press, 2002).

[19] J. Lass, D. Graf, F. Groitl, C. Kägi, R. Müller, R. Bürge, M. Schild, M. S. Lehmann, A. Bollhalder, P. Keller, M. Bartkowiak, U. Filges, F. Herzog, U. Greuter, G. Theidel, L. Testa, V. Favre, H. M. Rønnow, and C. Niedermayer, "Design and performance of the multiplexing spectrometer camea," (2020), arXiv:2007.14796 [physics.ins-det].

[20] J. Lass, H. Jacobsen, D. G. Mazzone, and K. Lefmann, SoftwareX **12**, 100600 (2020).

[21] Y. Zhang, H. Lu, X. Zhu, S. Tan, Q. Liu, Q. Chen, W. Feng, D. Xie, L. Luo, Y. Liu, H. Song, Z. Zhang, and X. Lai, Sci Rep **6**, 33613 (2016).

[22] M. C. Rahn, K. Kummer, A. Hariki, K.-H. Ahn, J. Kuneš, A. Amorese, J. D. Denlinger, D.-H. Lu, M. Hashimoto, E. Rienks, M. Valvidares, F. Haslbeck, D. D. Byler, K. J. McClellan, E. D. Bauer, J. X. Zhu, C. H. Booth, A. D. Christianson, J. M. Lawrence, F. Ronning, and M. Janoschek, Nat. Commun. **13**, 6129 (2022).
